# Herpes Simplex Virus 2 (HSV-2) Infected Cell Proteins Are among the Most Dominant Antigens of a Live-Attenuated HSV-2 Vaccine

**DOI:** 10.1371/journal.pone.0116091

**Published:** 2015-02-06

**Authors:** Joshua J. Geltz, Edward Gershburg, William P. Halford

**Affiliations:** Dept of Microbiology and Immunology, Southern Illinois University School of Medicine, Springfield, IL 62702, United States of America; UC Irvine Medical Center, UNITED STATES

## Abstract

Virion glycoproteins such as glycoprotein D (gD) are believed to be the dominant antigens of herpes simplex virus 2 (HSV-2). We have observed that mice immunized with a live HSV-2 *ICP0* ^-^ mutant virus, HSV-2 0ΔNLS, are 10 to 100 times better protected against genital herpes than mice immunized with a HSV-2 gD subunit vaccine (*PLoS ONE* 6:e17748). In light of these results, we sought to determine which viral proteins were the dominant antibody-generators (antigens) of the live HSV-2 0ΔNLS vaccine. Western blot analyses indicated the live HSV-2 0ΔNLS vaccine elicited an IgG antibody response against 9 or more viral proteins. Many antibodies were directed against infected-cell proteins of >100 kDa in size, and only 10 ± 5% of antibodies were directed against gD. Immunoprecipitation (IP) of total HSV-2 antigen with 0ΔNLS antiserum pulled down 19 viral proteins. Mass spectrometry suggested 44% of immunoprecipitated viral peptides were derived from two HSV-2 infected cells proteins, RR-1 and ICP8, whereas only 14% of immunoprecipitated peptides were derived from HSV-2’s thirteen glycoproteins. Collectively, the results suggest the immune response to the live HSV-2 0ΔNLS vaccine includes antibodies specific for infected cell proteins, capsid proteins, tegument proteins, and glycoproteins. This increased breadth of antibody-generating proteins may contribute to the live HSV-2 vaccine’s capacity to elicit superior protection against genital herpes relative to a gD subunit vaccine.

## Introduction

Herpes simplex virus 2 (HSV-2) infects more than 530 million people worldwide between the ages of 14 and 49 [1, 2], and >20 million individuals live with genital herpes disease that recurs more than once a year. Wild-type HSV-2 may cause severe infections in neonates [3, 4], and HSV-2-infected individuals are placed at ~3-fold higher risk for acquiring human immunodeficiency virus [5]. Hence, it is widely agreed that an effective HSV-2 vaccine is an important and unmet medical need.

Glycoprotein subunit vaccines represent the most widely studied approach to develop a safe and effective HSV-2 vaccine. Six clinical trials of HSV-2 glycoprotein D (gD-2) and/or glycoprotein B (gB-2) subunit vaccines have been conducted over the past 25 years, but have failed to prevent or reduce the symptoms of HSV-2 genital herpes [6, 7, 8, 9, 10, 11]. Our laboratory has investigated the potential of a live HSV-2 *ICP0* ^-^ mutant virus to address the unmet need for an effective HSV-2 vaccine. Our interest in the approach stemmed from the fact that HSV-1 *ICP0* ^-^ mutant viruses are exquisitely sensitive to repression by the innate interferon-α/β response [12, 13], and thus are profoundly attenuated in severe-combined immunodeficient (SCID) hosts [14]. The same is true of HSV-2 *ICP0* ^-^ mutant viruses [15].

In side-by-side comparisons, a live HSV-2 *ICP0* ^-^ mutant virus, HSV-2 0ΔNLS, elicited up to 100-times greater protection against HSV-2 genital herpes in mice and guinea pigs relative to animals immunized with a gD-2 subunit vaccine [16, 17]. Likewise, several whole HSV-2 vaccine approaches including dl-529 [18, 19], cJ2-gD2 [20], and killed HSV-2 + alum/MPL adjuvant [21] elicit superior protection against HSV-2 in animal models relative to gD-2 subunit vaccines. Although whole HSV-2 vaccines appear to be more effective than glycoprotein subunit vaccines, we lack a cohesive explanation as to why this should be the case. One possibility relates to the fact that the 302 amino acids of gD-2 included in subunit vaccines only corresponds to 0.8% of HSV-2’s proteome [22].

Our laboratory has proposed that increased antigenic breadth may explain, at least in part, the superior performance of whole HSV-2 viral vaccines relative to gD-2 subunit vaccines [23]. Specifically, if *antigenic breadth* equals the percentage (%) of an infectious agent’s proteome included in a vaccine, then the live HSV-2 0ΔNLS vaccine retains 99.3% of HSV-2’s antigenic breadth. This ~100-fold increase in antigenic breadth relative to gD-2 vaccines may contribute to the HSV-2 0ΔNLS vaccine’s capacity to elicit an ~400-fold reduction in HSV-2 vaginal shedding post-challenge relative to naïve controls. In contrast, gD-2-immunized animals shed ~4-fold less HSV-2 after challenge relative to naïve controls [17]. Mice and guinea pigs immunized with the live HSV-2 0ΔNLS vaccine generate ~40-fold higher levels of pan-HSV-2 IgG and ~20-fold higher levels of HSV-2-neutralizing antibody relative to animals immunized with a gD-2 vaccine [16, 17].

Just because HSV-2 0ΔNLS-immunized animals have high levels of HSV-2-specific antibody does not mean these antibodies contribute to protective immunity to HSV-2. Therefore, it is relevant to note that serum levels of pan-HSV-2 IgG antibody directly correlate with vaccine-induced protection against HSV-2 [16]. Moreover, naïve animals that receive an adoptive transfer of HSV-2 0ΔNLS antiserum possess significant (albeit incomplete) protection against HSV-2 challenge (Fig. 5 in Ref. [16]). Finally, our unpublished studies demonstrate that the live HSV-2 0ΔNLS vaccine elicits a robust virus-specific T-cell response in B-cell-deficient μMT mice, but 0ΔNLS-vaccinated μMT mice fail to effectively control HSV-2 vaginal challenge in the absence of virus-specific antibodies (unpublished data of W.P. Halford and K.J. Hasenkrug).

Given the potential role of HSV-2 0ΔNLS-induced antibodies in vaccine-induced protection against HSV-2, we were interested to identify the dominant antibody-generating (antigenic) proteins of the live HSV-2 0ΔNLS vaccine. Past studies of HSV-2 dl-529 or HSV-2 cJ2-D2 offer qualitative evidence that HSV-2 viral vaccines may elicit antibodies against many unspecified HSV-2 proteins in Western blots [20, 24]. However, an investigation has never been performed to positively identify one or more of the dominant antigens of a whole HSV-2 vaccine. Therefore, there is no published collection of methods that may be used to determine which of HSV-2’s 75 proteins are the dominant antibody-generators of a live HSV-2 vaccine. The current study was initiated to address this gap in knowledge.

We initially assumed that earlier studies of the humoral immune response to HSV-2 might delineate the immunodominance heirarchy of the antibody response to wild-type HSV-2. Although numerous studies between 1975 and 1990 used immunoblotting, ELISA, and immunoprecipitation techniques to test a hypothesis that HSV-2’s dominant antibody-generators (antigens) were virion glycoproteins [25, 26, 27], these analyses pre-dated the necessary scientific tools required to empirically determine what fraction of virus-specific antibodies were directed against HSV-2 glycoproteins relative to all of HSV-2’s proteins. For example, a complete HSV-2 genome sequence was not published until 1998 [28], and advanced proteomic tools did not emerge until after 2000. Nonetheless gB-2 and gD-2 emerged from this area of research in the 1980s as (1) the presumed dominant antigens of HSV-2 [27, 29] and (2) the leading candidates for an effective herpes subunit vaccine [30, 31, 32].

**Figure 1:**
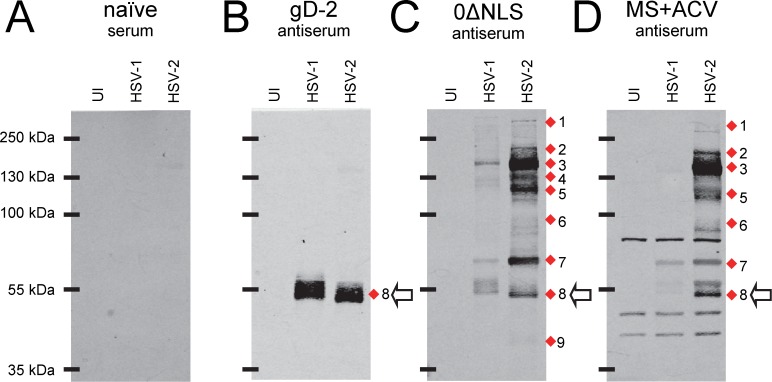
Western blot analysis to screen for candidate antibody-generating proteins of the live HSV-2 0ΔNLS vaccine. Representative Western blots of (UI) uninfected Vero cells or cells inoculated with 2.5 pfu/cell of HSV-1 KOS or HSV-2 MS incubated with 1:20,000 dilutions of serum from **(A)** mock-immunized mice (naïve) or mice immunized with **(B)** gD-2 + alum/MPL adjuvant, **(C)** HSV-2 0ΔNLS (*ICP0* ^-^) virus, or **(D)** an acyclovir-restrained HSV-2 MS infection (MS+ACV). Red diamonds (1–9) denote the positions of HSV-2 proteins most commonly targeted by mouse IgG antibodies, and the open arrow denotes the MW of gD-2.

**Figure 2:**
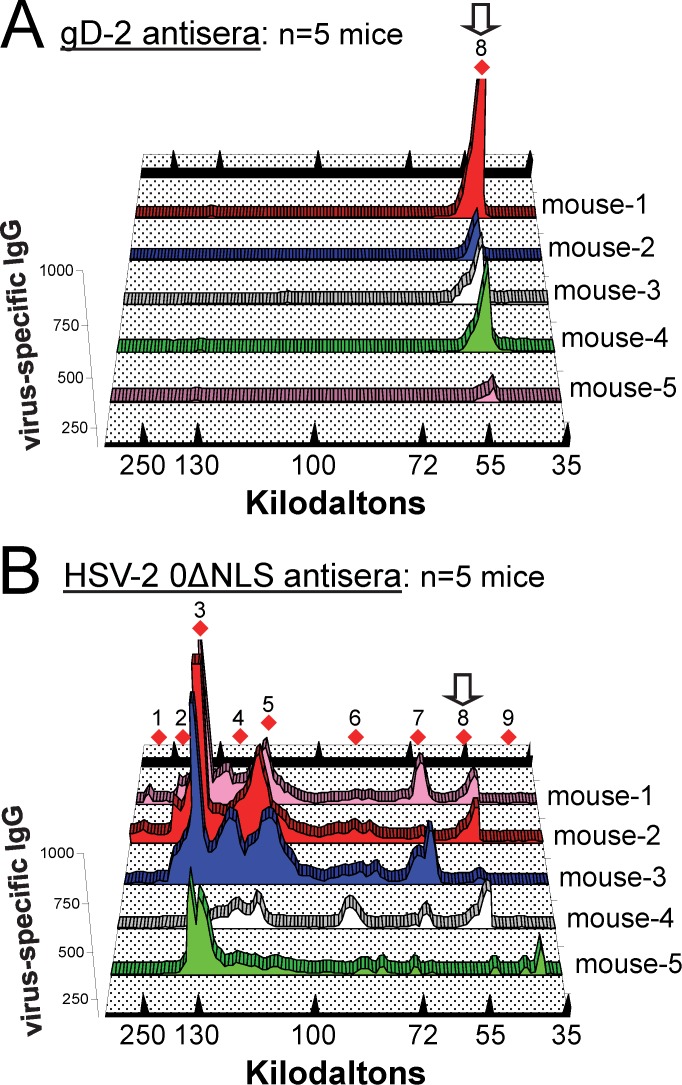
Virus-specific antibody binding to target HSV-2 proteins as a function of MW: gD-2 versus HSV-2 0ΔNLS antiserum. Three-dimensional line graphs summarize the relative intensity of IgG binding to HSV-2 proteins (y-axis) as a function of protein MW (x-axis) based on (**A**) sera from n = 5 gD-2-immunized mice (primary data shown in S1 Fig.) or **(B)** sera from n = 5 HSV-2 0ΔNLS-immunized mice (primary data shown in S1 Fig.). Red diamonds (1–9) denote positions of HSV-2 proteins commonly targeted by mouse IgG antibodies, and open arrows denote the MW of gD-2.

**Figure 3:**
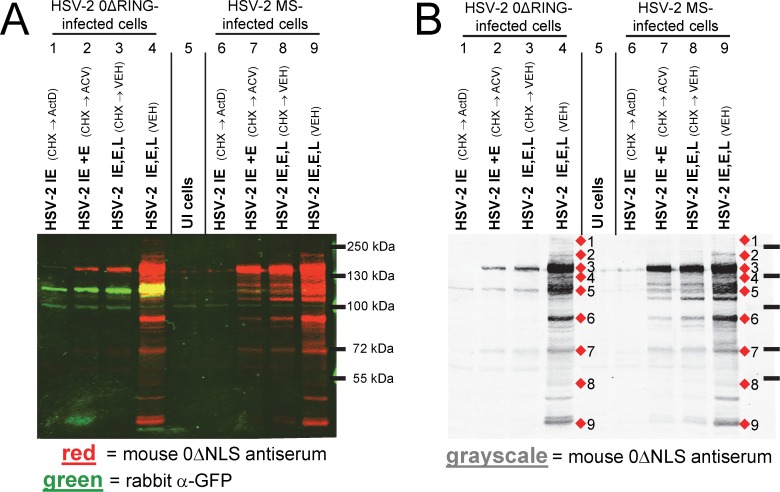
Cycloheximide-release analysis segregates candidate HSV-2 0ΔNLS antigens by IE, E, or L expression kinetics. (**A and B)** Western blot of Vero cells that were uninfected (UI) or were inoculated with 5 pfu per cell of HSV-2 0ΔRING or wild-type HSV-2 MS. Virus-infected cells were treated with cycloheximide (CHX) for 10 hours followed by 7 hours of treatment with actinomycin D (ActD; lanes 1 and 6); acyclovir (ACV; lanes 2 and 7); or no drug (VEH; vehicle; lanes 3 and 8). HSV-2 0ΔRING and HSV-2 MS-infected cells that were not drug-treated (lanes 4 and 9) were included as a control, and were harvested at 17 hours p.i. (**A**) Two-color analysis of HSV-2 proteins and GFP-tagged ICP0 (expressed by HSV-2 0ΔRING) labeled with 1:20,000 mouse α-0ΔNLS antiserum (red signal) and 1:5,000 rabbit α-GFP antiserum (green signal). (**B)** Grayscale representation of mouse IgG (in 0ΔNLS antiserum) binding to HSV-2 proteins.

**Figure 4:**
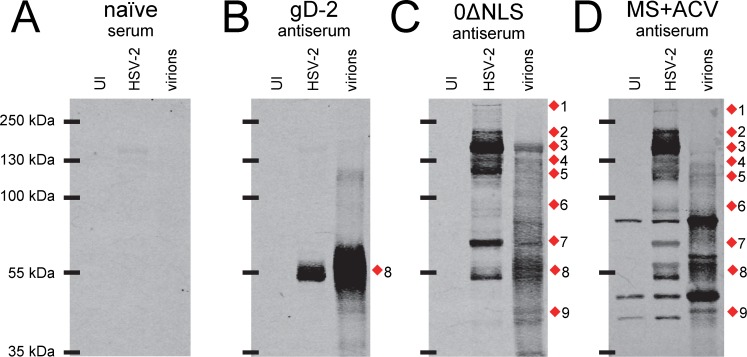
Western blot analysis of purified HSV-2 virions segregates candidate HSV-2 0ΔNLS antigens into infected cell proteins versus virion proteins. Representative Western blots of (UI) uninfected Vero cells, total HSV-2-infected cell proteins (MOI = 2.5), or sucrose-gradient-purified HSV-2 virions incubated with 1:20,000 dilutions of serum from **(A)** a mock-immunized mouse (naïve) or mice immunized with **(B)** gD-2 + alum/MPL adjuvant, **(C)** HSV-2 0ΔNLS, or **(D)** an acyclovir-restrained HSV-2 MS infection (MS+ACV). Red diamonds (1–9) denote the positions of viral proteins in total HSV-2-infected cell samples most commonly targeted by mouse IgG antibodies.

**Figure 5:**
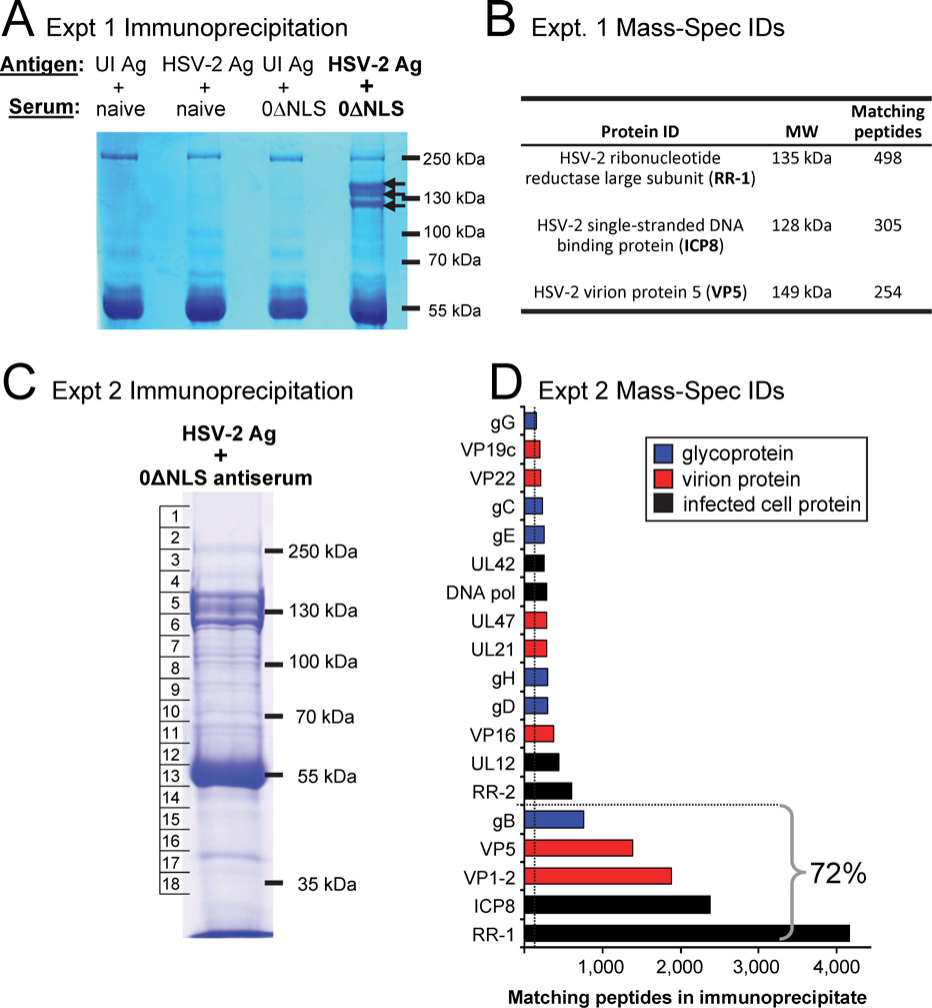
Immunoprecipitation-mass spectrometry (IP-mass spec) analysis as a tool to screen antibody specificities in HSV-2 0ΔNLS antiserum. **(A-B)** IP-mass spec experiment #1. Uninfected Vero cell proteins (UI Ag) or HSV-2 MS-infected cell proteins (HSV-2 Ag) were resuspended in a NP40-based buffer containing 150 mM NaCl and were incubated with 2% naïve mouse serum or 2% mouse 0ΔNLS-antiserum for 2 hours followed by overnight incubation with Protein A/G agarose beads. **(A)** Coomassie-blue stained polyacrylamide gel of immunoprecipitates formed by HSV-2 Ag + mouse 0ΔNLS antiserum versus three negative-control immunoprecipitation reactions. Black arrows denote three protein species pulled down by 0ΔNLS antiserum that were not present in controls. **(B)** Identity of proteins excised from the gel (panel A), as determined by MALDI-TOF mass spectrometry. **(C-D).** IP-mass spec experiment #2. **(C)** Coomassie-blue stained polyacrylamide gel of immunoprecipitates formed by HSV-2 MS-infected cell proteins (HSV-2 Ag) following incubation with 1% mouse 0ΔNLS-antiserum and Protein A/G agarose beads. The entire lane of the gel was analyzed by MALDI-TOF mass spectrometry after being cut into 18 equivalent sized slices (denoted by boxes 1–18); slice-by-slice mass spectrometry identification results for the five most abundant HSV-2 proteins are shown in S3 Fig. **(D)** Number of peptide matches per positively identified HSV-2 protein. A total of 14,729 peptides were identified by mass spectrometry as being derived from 19 HSV-2 proteins that met our inclusion criteria, which were that a “positive identification” should (1) contribute >1% to the total pool of positive HSV-2 peptides (i.e., >147 peptides); (2) have >30% of its peptides recovered from 3 consecutive gel slices at the protein’s expected MW (e.g., S3 Fig.); (3) have >25% of its protein sequence represented were detected by the mass spectrometer, and should (4) yield 10 or more unique peptides. Seventy-two percent of the positive HSV-2 peptides in immunoprecipitates were derived from the 5 most dominant proteins identified; namely, RR-1, ICP8, VP1–2, VP5, and gB.

**Figure 6:**
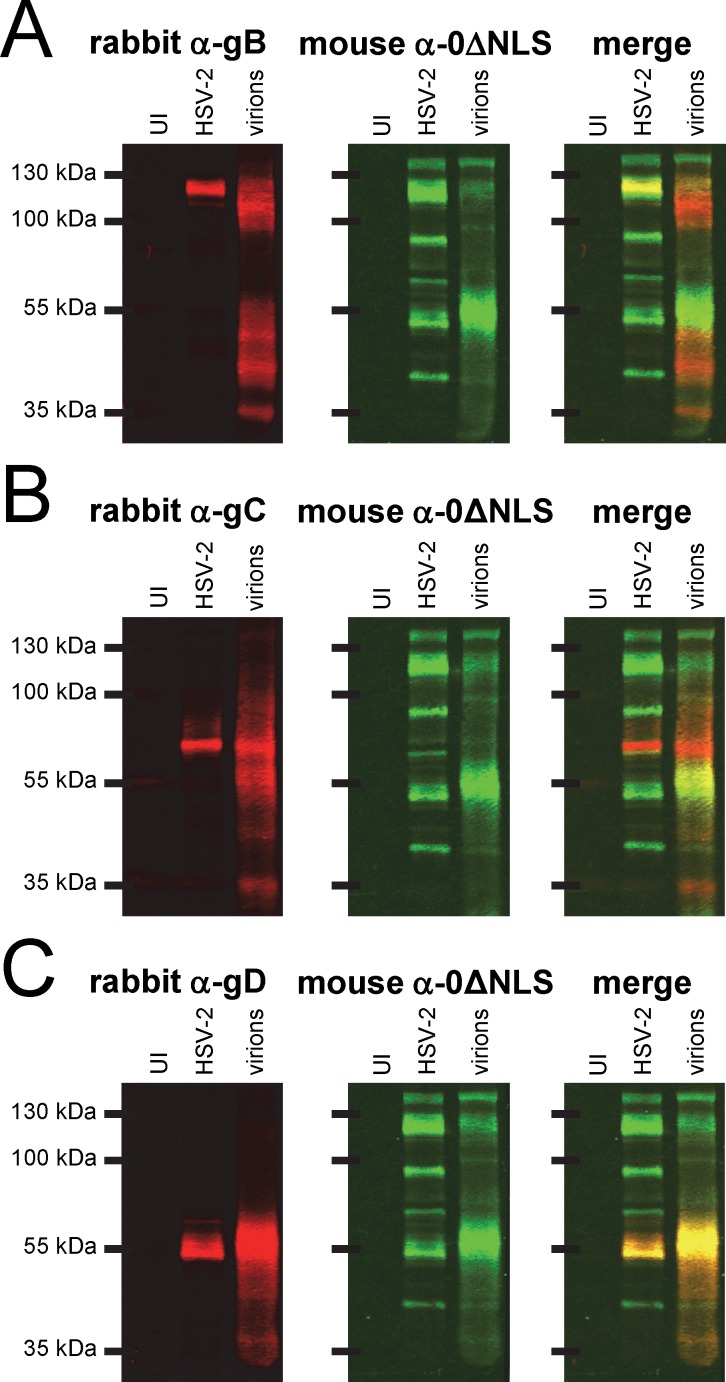
Two-color Western blot: mouse HSV-2 0ΔNLS antiserum versus rabbit antisera against HSV-2 glycoproteins B, C, and D. Western blots of (UI) uninfected Vero cells, total HSV-2-infected cell proteins (MOI = 2.5), or sucrose-gradient-purified HSV-2 virions were incubated with a 1:20,000 dilution of mouse HSV-2 0ΔNLS antiserum (green signal = mouse IgG) and 1:10,000 dilutions of rabbit antisera specific for **(A)** HSV-2 gB, **(B)** HSV-2 gC, or **(C)** HSV-2 gD (red signal = rabbit IgG).

**Figure 7:**
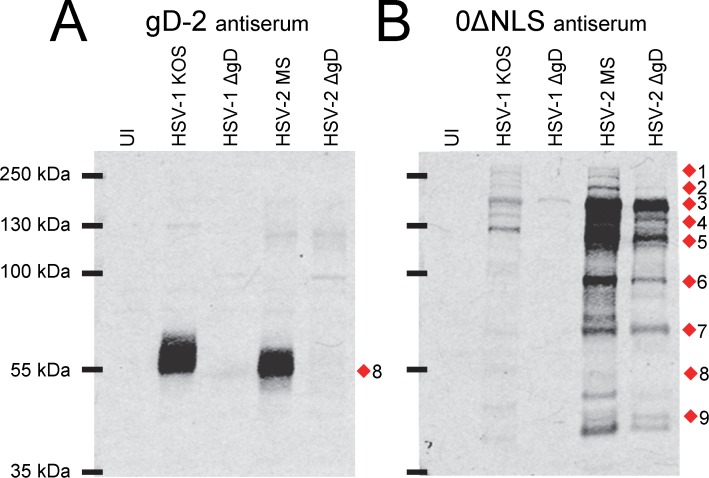
Western blot analysis of HSV gD-antigen-deletion mutants: effect on antibody-binding targets of gD-2 antiserum versus HSV-2 0ΔNLS antiserum. Western blots of (UI) uninfected Vero cells or cells inoculated with 5 pfu/cell of HSV-1 KOS, a HSV-1 ΔgD virus (KOS-gD6), HSV-2 MS, or a HSV-2 ΔgD virus (HSV-2 ΔgD-BAC) incubated with 1:20,000 dilutions of serum from mice immunized with **(A)** gD-2 + alum/MPL adjuvant or **(B)** HSV-2 0ΔNLS. Red diamonds (1–9) denote the positions of viral proteins most commonly targeted by mouse IgG antibodies.

**Figure 8:**
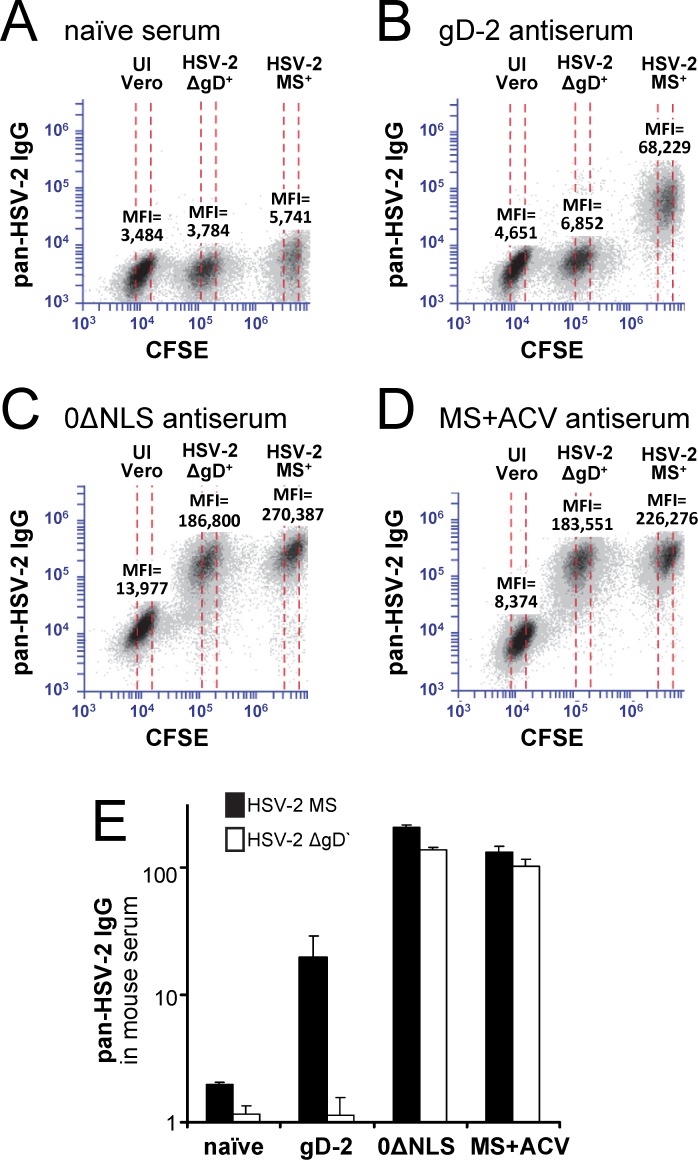
Flow cytometric analysis of HSV gD-antigen-deletion mutants: effect on antibody-binding targets of gD-2 antiserum versus HSV-2 0ΔNLS antiserum. Three-population cytometric analysis comparing IgG antibody-binding to a mixture of uninfected (UI) Vero cells versus Vero cells inoculated with HSV-2 ΔgD-BAC or HSV-2 MS. Each cell population was dispersed, differentially labeled with 0, 0.45, or 6 μM CFSE, fixed, permeabilized, and combined for antibody staining and flow cytometry. **(A—D)** Mixed populations of test cells were incubated with 1:6,000 dilutions of serum from **(A)** a naïve mouse or a mouse immunized with **(B)** gD-2 + alum/MPL, **(C)** HSV-2 0ΔNLS, or **(D)** an acyclovir-restrained HSV-2 MS infection (MS+ACV). Pan-HSV-2 IgG binding (y-axes) was detected using APC-labeled goat anti-mouse IgG secondary, and was measured in three gates (dashed columns) at the center of the CFSE-negative, CFSE ^lo^, and CFSE ^hi^ populations to compare IgG binding to UI cells, HSV-2 ΔgD^+^ cells, versus HSV-2 MS^+^ cells, respectively. **E.** Mean ± sem of pan-HSV-2 IgG levels in n = 5 mice per immunization group, as measured by ***i.*** the increase in mean fluorescent intensity (ΔMFI) of IgG bound to HSV-2 MS^+^ cells relative to UI cells, and ***ii.*** the ΔMFI of IgG bound to ΔgD^+^ cells relative to UI cells.

**Figure 9:**
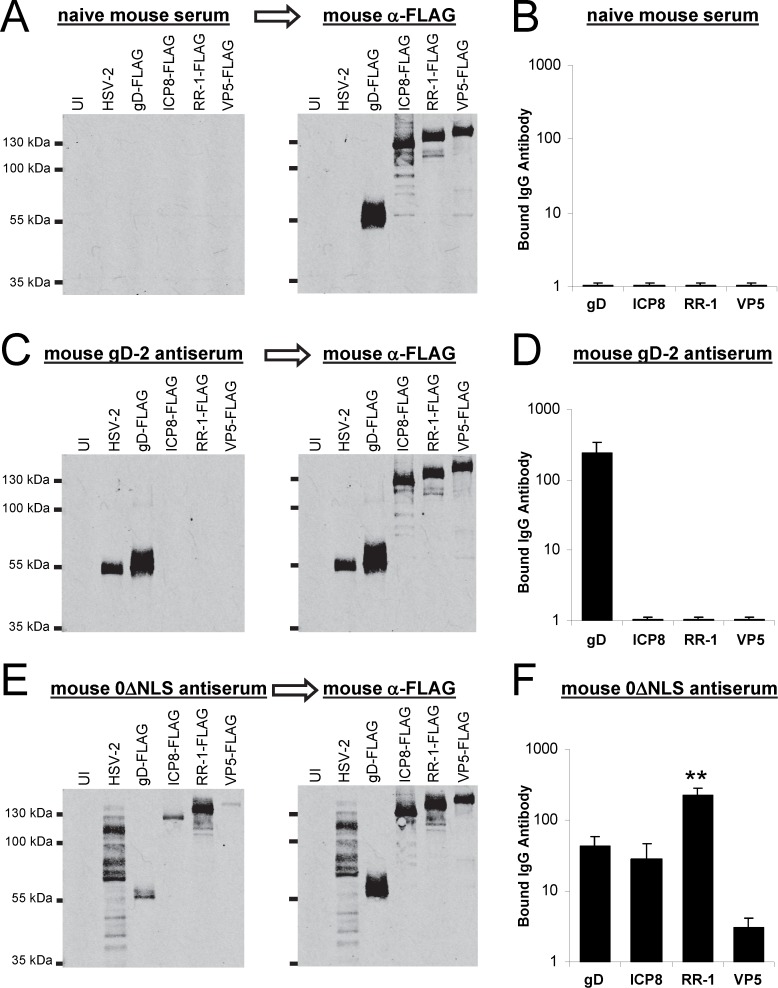
Cell lines expressing epitope-tagged HSV-2 antigens: relative abundance of gD-, ICP8-, RR-1-, and VP5-specific antibodies in HSV-2 0ΔNLS antiserum. Western blots of (UI) uninfected Vero cells, cells inoculated with 2.5 pfu/cell of HSV-2 MS, or Vero cell lines that stably express the following, recombinant HSV-2 proteins: gD-FLAG, ICP8-FLAG, RR-1-FLAG, or VP5-FLAG incubated with 1:20,000 dilutions of serum from (**A**) naïve mice or (**C, E**) mice immunized with **(C)** gD-2 + alum/MPL adjuvant or **(E)** HSV-2 0ΔNLS. Following incubation with mouse serum, blots were rinsed and re-probed with mouse α-FLAG antibody to validate the relative amount of FLAG-tagged HSV-2 protein on each blot. (**B, D, F**) Normalized amount of IgG antibody bound to recombinant gD, FLAG, RR-1, or VP5 on blots incubated with (**B**) naïve mouse serum (n = 3), (**D**) gD-2 antiserum (n = 3), or (**F**) 0ΔNLS antiserum (n = 6). Levels of bound IgG antibody were normalized to account for blot-to-blot variance in the relative amount of each target based on the relative amount of α-FLAG antibody that bound each recombinant protein. In panel F, ‘**’ denotes that IgG antibody in 0ΔNLS antiserum bound RR-1 to significantly greater levels than gD, ICP8, or VP5 (p<0.001, one-way ANOVA and Tukey’s post-hoc-t-test).

In the absence of a clear precedent, we chose to develop a novel collection of methods for the purpose of identifying the dominant antibody-generating proteins of the live HSV-2 0ΔNLS vaccine. We outline the logic of this process, as follows. First, Western blot analysis was used to identify the molecular weight (MW) of candidate antibody-generating proteins (Figs. 1 and 2). Second, the list of potential antibody-generating HSV-2 proteins was further refined based on the (1) kinetics with which the HSV-2 antigen was expressed (Fig. 3) and (2) whether the HSV-2 antigen was an infected-cell protein or virion structural component (Fig. 4). Third, immunoprecipitation-mass spectrometry (IP-mass spec) provided a powerful screening tool to identify candidate HSV-2 antigens (Fig. 5). While the results of these 4 screening approaches could be cross-referenced to identify likely dominant antigens, each method had caveats and limitations that called into question which putative “dominant antigens” were real and which were artefacts. Hence, we explored three independent methods that might be used to positively identify one or more *bona fide* antibody-generating proteins of the live HSV-2 0ΔNLS vaccine; namely, ***a.*** two-color Western blot analysis, ***b.*** tests with antigen-deleted HSV-2 mutants, and ***c.*** tests with cell lines that expressed individual, epitope-tagged HSV-2 antigens (Figs. 6–9).

The results presented below offer the first detailed and unbiased characterization of the IgG antibody response to a live-attenuated HSV-2 vaccine in the post-genomic era. Scores of tests using this collection of methods led us to two central conclusions: ***1.*** the serum IgG antibody response of mice immunized with the live HSV-2 0ΔNLS vaccine is directed against 9 to 19 different viral proteins; and ***2.*** two of the major antigens of the live HSV-2 0ΔNLS vaccine are not virion glycoproteins, but are the infected cell proteins ICP8 and RR-1. ICP8 is HSV-2’s single-stranded DNA-binding protein [33], and RR-1 is the large subunit of HSV-2’s ribonucleotide reductase [34]. These results raise serious questions about the accuracy of a long-standing assertion that most of HSV-2’s dominant antigens are virion glycoproteins [27, 29].

## Results

### HSV-2 0ΔNLS elicits a polyclonal IgG response against at least 9 viral proteins

In a prior study, mice were immunized on Days 0 and 30 with ***i.*** culture medium (naïve), ***ii.*** live HSV-2 0ΔNLS, ***iii.*** adjuvanted glycoprotein D-2 (gD-2), or ***iv.*** wild-type HSV-2 MS where infection was restrained with acyclovir (MS+ACV), and sera were collected on Day 60 [17]. In the current study, mouse sera were retrospectively analyzed to identify one or more viral proteins that were dominant antibody-generators of the live HSV-2 0ΔNLS vaccine.

Mouse sera were tested against Western blots containing uninfected (UI) Vero cells or cells inoculated with HSV-1 strain KOS or HSV-2 strain MS (Fig. 1). Naïve serum did not exhibit specific reactivity against viral proteins (Fig. 1A). Serum from a gD-2-immunized mouse contained high levels of IgG that bound HSV-2’s ~55 kDa gD-2 protein, and extensively cross-reacted with HSV-1’s gD protein (Fig. 1B). Serum from a HSV-2 0ΔNLS-immunized mouse contained IgG antibodies that bound many HSV-2 proteins, which ranged in molecular weight (MW) from 40 to >250 kDa (Fig. 1C). Specifically, IgG antibody bound HSV-2 proteins with peak intensities at MWs of ~320, 215, 140, 130, 115, 90, 70, 55, and 40 kDa (Fig. 1C; bands 1—9). Serum from a mouse immunized with an acyclovir-restrained HSV-2 MS infection (MS+ACV) exhibited a similar IgG response to multiple HSV-2 proteins, but two differences were noted (Fig. 1D). The serum from “MS + ACV”-immunized mice possessed high levels of IgG directed against three cellular proteins present in UI cells and virus-infected cells, whose MWs were ~80, 45, and 40 kDa (Fig. 1D). In addition, the ~130 kDa HSV-2 protein bound by 0ΔNLS antiserum (i.e., band 4) was not observed when a blot was incubated with MS+ACV antiserum (Fig. 1D).

HSV-1 and HSV-2 share co-linear genomes that encode ~75 homologous viral proteins, and many of these homologous proteins share ~90% amino acid identity. Nonetheless, HSV-1 and HSV-2 represent two different serotypes of the same virus, and thus pre-existing adaptive immunity to HSV-1 does not protect against HSV-2 infection [35, 36]. Due to the serotype-specific nature of the immune response to HSV-1 versus HSV-2, Western blots have been used for decades to differentiate whether humans are infected with HSV-1 or HSV-2 [37, 38]. Consistent with the serotype-specific response observed in humans, mice immunized with the live HSV-2 0ΔNLS vaccine exhibited high levels of antibodies that efficiently bound proteins encoded by HSV-2 strain MS in Western blots, but which only weakly cross-reacted with the 75 homologous proteins encoded by HSV-1 (Fig. 1C).

The results presented in Fig. 1 were based on a single mouse per immunization group, and provided no basis to assess the animal-to-animal variance within immunization groups. To address this limitation, the sera of n = 5 mice per immunization group were tested against equivalent Western blots (S1 Fig.). To increase the opportunity to observe heterogeneity in the antibody response, Day 60 pre-challenge sera chosen for this retrospective analysis from mice that exhibited a range of levels of vaccine-induced protection against HSV-2 vaginal challenge (Fig. 4 in Ref. [17]). IgG in the sera of gD-2-immunized mice was consistently directed against an ~55 kDa HSV-2 protein (S1 Fig.). IgG in the sera of n = 5 HSV-2 0ΔNLS-immunized mice consistently bound 6 or more HSV-2 proteins whose MWs ranged from 40 to >250 kDa (bands 1—9 in S1 Fig.). Likewise, IgG in the sera of n = 5 MS+ACV-immunized mice consistently bound 4 or more HSV-2 proteins whose MWs ranged from 50 to >250 kDa (bands 1—8 in S1 Fig.). Unexpectedly, all MS+ACV-immunized mice exhibited a prominent antibody response against cellular proteins that were 40, 45, or 80 kDa in size (S1 Fig.). These results suggested the live HSV-2 0ΔNLS vaccine elicited a polyclonal IgG antibody response against many HSV-2 antigens other than gD-2, and this antibody response was similar in complexity to that elicited by an ACV-restrained, wild-type HSV-2 MS infection.

### The most dominant antigens of HSV-2 0ΔNLS are between 110 and 165 kDa in size

Western blot images may vary slightly in size and shape for a variety of technical reasons. The fact that protein bands and MW markers shift in both x- and y-coordinates makes it difficult to visually compare blots with any confidence that trends observed (or missed) are unaffected by the human observer. Hence, we developed a computational method to allow the quantitative data obtained by infrared Western blot scanning to be transferred into a x-, y- data set where the x-coordinate represents protein MW in the HSV-2 protein lane of a gel and the y-coordinate represents the intensity of IgG antibody binding at that position (Fig. 2). In each gel, the protein MW markers were used to convert “distance migrated” into “log (kDa)” per the equation p = *a* ● *e* ^*k* ● *x*^ where p = predicted log (kDa), x = observed distance of migration, and ‘a’ and ‘k’ were constants dictated by the positions of protein MW markers and were solved by the method of least squares (described in Methods). The key to standardizing blot-to-blot results lay in the introduction of two correction factors for (1) “vertical offset” to compensate for blot-to-blot variance in the x-position of the highest MW marker (250 kD) and (2) “compression” which compensated for subtle variance (± 5%) in gel-to-gel stretching between blots. Hence, it was possible to normalize the distance migrated of the protein MW markers such that the 250 kDa and 55 kDa markers were always set to the same x-coordinates, and the distance of migration of all other protein MW markers could be scaled accordingly.

These computational methods were applied to analyze IgG antibody binding to HSV-2 Western blots incubated with serum from n = 5 mice immunized with gD-2 or n = 5 mice immunized with the HSV-2 0ΔNLS vaccine (S1 Fig.). In 5 of 5 gD-2-immunized mice, antisera bound a single HSV-2 protein whose peak intensity occurred at ~55 kDa (Fig. 2A). In contrast, in 5 of 5 HSV-2 0ΔNLS-immunized mice, antisera contained a polyclonal population of IgG antibodies directed against multiple HSV-2 proteins of widely varying MWs (Fig. 2B). Peaks of IgG antibody binding to HSV-2 proteins occurred at approximate MWs of 320, 215, 140, 130, 115, 90, 70, 55, and 40 kDa (Bands 1—9 in Fig. 2B). Band 8 coincided with the MW of gD-2. Comparison of peak intensities of IgG binding revealed that 9 ± 6% of the IgG in 0ΔNLS antisera bound the ~55 kDa protein relative to the summated intensity of IgG binding to all 9 proteins (Fig. 2B; Table 1). In contrast, the same peak intensity analysis suggested that the dominant antigens of the HSV-2 0ΔNLS vaccine were 140, 130, and 115 kDa in size, and collectively bound 71 ± 20% of the virus-specific IgG in 0ΔNLS antiserum (Fig. 2B; Table 1).

**Table 1:** Intensity of 0ΔNLS antiserum IgG binding of HSV-2 Proteins by MW: Peak Analysis.

**Protein**	**MW (kDa)**	**% IgG bound**
**1 ^a^**	320 ^b^	**0.8 ± 0.4 ^c^**
**2**	215	**4 ± 2**
**3**	140	**44 ± 10**
**4**	130	**13 ± 7**
**5**	115	**14 ± 3**
**6**	90	**6 ± 4**
**7**	70	**8 ± 3**
**8**	55	**9 ± 6**
**9**	**40**	**2 ± 2**

^a^ Numbers correspond to protein bands denoted by red diamonds in Figs. 1 and 2.

^b^ Apparent molecular weight of proteins bound by IgG in mouse 0ΔNLS antiserum.

^c^ Mean ± sem intensity of IgG binding to each of 9 HSV-2 proteins, as calculated from the percentage of “peak signal associated with each of the 9 bands in the HSV-2 lane” relative to the “summation of peak signals in all 9 protein positions on the same blot.” The mean ± sem of these values were calculated from n = 5 blots incubated with the serum of 1 of 5 mice immunized with HSV-2 0ΔNLS (S1 Fig.).

A more conventional, area-under-the-curve analysis supported a similar conclusion (Table 2). Specifically, 11 ± 4% of IgG antibodies in HSV-2 0ΔNLS antisera bound HSV-2 proteins between 35 and 65 kDa, whereas 60 ± 5% of IgG antibodies in HSV-2 0ΔNLS antisera bound HSV-2 proteins between 110 and 165 kDa (Table 2). Hence, densitometric analysis of Western blots (S1 Fig.) suggested that HSV-2 0ΔNLS’s most dominant antigens were viral proteins in the size range of 110 to 165 kDa.

**Table 2:** Intensity of 0ΔNLS antiserum IgG binding of HSV-2 Proteins by MW: Area-Under-Curve Analysis.

**Protein MW range**	**% IgG bound**
165—350 ^a^	9 ± 2 ^b^
110—165	60 ± 5
65—110	22 ± 3
35—65	11 ± 4

^a^ Protein molecular weight ranges analyzed for IgG binding intensity by calculating the area under the curve for each lane profile shown in S2 Fig.

^b^ Mean ± sem intensity of IgG binding in the indicated MW range in the HSV-2 lane of blots shown in Fig. 2. For each measurement, percentage of “summated signal for MW range” was calculated relative to the “summated signal for the entire HSV-2 lane.” The mean ± sem values were calculated from n = 5 Western blots incubated with serum of mice immunized with the HSV-2 0ΔNLS vaccine (S1 Fig.).

### The dominant 140 kDa antigen of HSV-2 0ΔNLS is a viral early protein

More than 10 viral proteins migrate in the size range of 110 to 165 kDa including HSV-2’s immediate-early (IE) transcriptional regulator, ICP0. A cycloheximide (CHX)-release experiment was performed to determine if the likely identity of any of the antibody-generating proteins of the live HSV-2 0ΔNLS vaccine might be clarified based on whether these viral proteins were expressed with IE, early (E), or late (L) kinetics (Fig. 3).

To this end, Vero cells were treated with CHX to block protein translation, and were inoculated with 5 pfu per cell of a HSV-2 *ICP0* ^-^ virus, HSV-2 0ΔRING, or wild-type HSV-2 MS. By 10 hours post-inoculation, HSV-2 IE mRNAs had accumulated and the CHX block was replaced with ***i.*** actinomycin D (Act D) which allows only viral IE proteins to accumulate (Fig. 3A, lanes 1 and 6); ***ii.*** acyclovir (ACV) which allows viral IE and E proteins to accumulate (Fig. 3A, lanes 2 and 7); or ***iii.*** culture medium lacking any inhibitor (vehicle; VEH; Fig. 3A, lanes 3 and 8). This panel of HSV-2 protein samples was blotted, and incubated with rabbit α-GFP antiserum and mouse α-HSV-2 0ΔNLS serum (Fig. 3A).

The *ICP0* ^-^ virus, HSV-2 0ΔRING, expressed high levels of a GFP-tagged ICP0 protein with IE kinetics, which was readily detected by the rabbit α-GFP antibody (green band, lane 1, Fig. 3A). In contrast, mouse 0ΔNLS antiserum failed to react with wild-type ICP0 in HSV-2 MS-infected cells that received an equivalent CHX → Act D treatment (lane 6, Fig. 3A). IgG antibodies in 0ΔNLS antiserum bound an ~140 kDa E protein that was expressed to high levels when CHX-treated cells were released into ACV (lanes 2 and 7, Fig. 3A).

Grayscale analysis of the IgG binding profile of mouse 0ΔNLS antiserum, which permitted better visualization of bands, revealed three notable features (Fig. 3B). First, mouse 0ΔNLS antiserum contained detectable levels of IgG antibody against the “GFP tag” in the mutant ICP0 protein expressed by HSV-2 0ΔRING (lane 1, Fig. 3B), but did not contain detectable levels of IgG against the wild-type ICP0 expressed by HSV-2 MS (lane 6 in Fig. 3B). Second, protein bands 1, 2, 8, and 9 appeared to be expressed with late kinetics. Third, lanes 7, 8, and 9 provided three adjacent HSV-2 MS-infected cell samples, which allowed clear visualization of IgG antibody binding to numerous HSV-2 protein species other than bands 1—9 (Fig. 3B). Hence, the serum of this 0ΔNLS-immunized mouse appeared to contain IgG antibodies directed against at least 14 distinct HSV-2 protein species.

This kinetic analysis provided a clue as to the possible identity of the dominant ~140 kDa E antigen of the HSV-2 0ΔNLS vaccine. This was highly reminiscent of the ~160 kDa “AG-4” antigen described in the early 1980s, which was precipitated by the serum of >90% of persons infected with HSV-2 [39], and which contained an abundance of two HSV-2 E proteins, RR-1 (UL39) and ICP8 (UL29) [34, 40]. Further studies were conducted to determine if, in fact, one or more of the most dominant antigens of the HSV-2 0ΔNLS vaccine was an infected cell protein rather than the virion glycoproteins that are often assumed to be the dominant targets of the antibody response to HSV-2 [11, 27].

### One or more dominant antigens of HSV-2 0ΔNLS are infected-cell proteins

HSV-2-infected cells proteins such as RR-1 and ICP8 are, by definition, only found in virus-infected cells, and are not incorporated into the structure of HSV-2 virions. An experiment was conducted to determine if some of the antigens of the live HSV-2 0ΔNLS vaccine were infected cell proteins rather than structural components of HSV-2 virions. To this end, IgG antibody reactivity was compared in Western blots containing total HSV-2-infected cell proteins versus sucrose gradient-purified HSV-2 virions (Fig. 4). Serum from a naïve mouse established that non-specific IgG binding to Western blots was negligible (Fig. 4A). Serum from a gD-2-immunized mouse contained IgG antibodies that reacted with an ~55 kDa gD-2 protein present in HSV-2-infected cells, which was enriched by ~4-fold in HSV-2 virions (Fig. 4B). The serum of a HSV-2 0ΔNLS-immunized mouse reacted strongly with infected cell proteins of 140, 120, and ~70 kDa that were not enriched in HSV-2 virions (bands 3, 5, and 7 in Fig. 4C). In contrast, a sub-population of IgG antibodies bound a protein slightly larger than 55 kDa that was enriched in HSV-2 virions, and which corresponded to the predicted size of the mature form of gD-2 (Fig. 4C). IgG antibodies in 0ΔNLS antiserum bound protein species that were prominent in purified HSV-2 virions, but which were inapparent in virus-infected cells (Fig. 4C). Hence, enrichment of HSV-2 virions presented another potential opportunity to explore the complexity of the antibody response to the HSV-2 0ΔNLS vaccine.

A mouse immunized with an ACV-restrained HSV-2 MS infection exhibited a similar pattern of IgG antibody reactivity against ~140, 115, and 70 kDa infected cell proteins that were not enriched in HSV-2 virions (bands 3, 5, 7 in Fig. 4D). Like HSV-2 0ΔNLS antiserum, IgG antibodies in the serum of MS+ACV-immunized mice specifically bound an ~55 kDa protein that was enriched in HSV-2 virions, and which was presumably gD-2 (Fig. 4D). Intriguingly, serum from MS+ACV-immunized mice contained high levels of IgG antibodies against ~45 and 80 kDa cellular proteins that were ***1.*** present in UI Vero cells and which were ***2.*** enriched by 4-fold in purified HSV-2 virions (Fig. 4D). Independent Western blots suggested these ~45 and 80 kDa cellular proteins were unlikely to be non-specific cellular contaminants, as the glycolytic enzyme GAPDH was abundant in virus-infected cells but undetectable in purified virions (not shown). Therefore, these ~45 and 80 kDa cellular proteins may represent host proteins that are incorporated into the tegument of HSV-2 virions, and which may also serve as autoantigens. However, this tangential observation was not further pursued.

These findings suggested that one of the most dominant antigens of the HSV-2 0ΔNLS vaccine was an 140 kDa E protein that was a virus infected-cell protein rather than a glycoprotein or other HSV-2 virion component (band 3 in Fig. 4C). To verify the reproducibility of this finding, the sera of n = 5 mice per immunization group were tested against similar Western blots (S2 Fig.). HSV-2-specific IgG antibody in gD-2-immunized mice was consistently directed against an ~55 kDa protein that was enriched in purified virions by 4.2 ± 0.8-fold per densitometric analysis (S2 Fig.). In contrast, the sera of 5 of 5 mice immunized with the HSV-2 0ΔNLS vaccine contained high levels of antibody against an ~140 kDa infected-cell protein (S2 Fig.). Likewise, 4 of 5 MS+ACV-immunized mice contained high levels of antibody against an ~140 kDa viral proteins that was not enriched in purified HSV-2 virions (S2 Fig.). Collectively, the data presented in Figs. 3 and 4 suggested that the dominant ~140 kDa antigen of the live HSV-2 0ΔNLS vaccine was not a virion glycoprotein, but rather was an infected cell protein that was expressed with E kinetics.

### Identification of viral proteins targeted by HSV-2 0ΔNLS antiserum: IP-mass spec analysis

The dominant antigens of the HSV-2 0ΔNLS vaccine appeared to include at least one viral E protein whose MW was ~140 kDa (Fig. 1C, Table 1). A series of immunoprecipitation-mass spectrometry (IP-mass spec) experiments was conducted to screen for the potential identity of one or more of the HSV-2 0ΔNLS vaccine’s dominant antigens.

Total protein from uninfected Vero cells (UI Ag) or HSV-2-infected Vero cells (HSV-2 Ag) was immunoprecipitated with naïve serum or mouse 0ΔNLS antiserum, and immunoprecipitates were electrophoresed and stained with Coomassie Blue (Fig. 5A). Three control immunoprecipitation (IP) reactions defined the background of the assay; namely, ***i.*** UI Ag + naïve serum; ***ii.*** HSV-2 Ag + naïve serum; and 3. UI Ag + 0ΔNLS antiserum (Fig. 5A). In contrast, HSV-2 Ag + 0ΔNLS antiserum precipitated three proteins in the 100 to 150 kDa size range (arrows in Fig. 5A). These bands were excised and subjected to in-gel trypsinization and matrix-assisted laser desorption ionization time of flight (MALDI-TOF) mass spectrometry (mass spec). This analysis yielded ~250 to 500 independent peptides per protein that matched HSV-2’s virion protein 5 (VP5), infected-cell protein 8 (ICP8), and ribonucleotide reductase-1 (RR-1) (Fig. 5B). VP5 is HSV-2’s major capsid protein and is encoded by the UL19 gene. ICP8 and RR-1 are infected cell proteins, where ICP8 is HSV-2’s single-stranded DNA binding protein and RR-1 is the large subunit of HSV-2’s ribonucleotide reductase, which are encoded by the viral UL29 and UL39 genes, respectively (Fig. 5B).

A second and more comprehensive IP-mass spec experiment was performed. HSV-2-infected Vero cell proteins were immunoprecipitated with mouse 0ΔNLS antiserum, electrophoresed, and visualized with a Coomassie blue stain (Fig. 5C). The entire gel lane was sub-divided into 18 slices and subjected to in-gel trypsinization, and MALDI-TOF mass spectrometry (S3 Fig.). A total of 43 HSV-2 proteins were detected in immunoprecipitates, but only 19 viral proteins met our minimum criteria for “positive identification,” as defined in the Methods section. Two of the primary criteria that excluded 23 HSV-2 proteins from further consideration were they ***1.*** contributed less than 1% to the total pool of positively identified viral peptides and/or ***2.*** too few peptide hits were observed in consecutive gel slices at the correct MW of the identified protein (S3 Fig.).

Of the 14,729 viral peptides that met our inclusion criteria, 72% were derived from the five most dominant proteins in immunoprecipitates; namely, RR-1, ICP8, VP1–2, VP5, and gB-2 (Fig. 5D). Six glycoproteins were immunoprecipitated by 0ΔNLS antiserum; these were gB-2, gD-2, gH-2, gE-2, gC-2, and gG-2 which represented 14% of the HSV-2 peptides (Fig. 5D). The results of these two-IP mass spec analyses were consistent with initial Western blot results, and suggested that RR-1 and ICP8 were ~130 and 140 kDa infected-cell proteins targeted by the IgG antibody response of HSV-2 0ΔNLS-immunized mice (Fig. 2B, Table 1).

### Confirmation Method 1: two-color Western blot analysis

Western blot and IP-mass spec experiments offered valuable screening tools to focus on which of HSV-2’s 75 proteins might serve as dominant antibody-generators of the live HSV-2 0ΔNLS vaccine. However, each method had its caveats, and thus we explored several methods that might be used to confirm the identity of dominant antigens of the HSV-2 0ΔNLS vaccine. The first method considered was two-color Western blot analysis.

IP-mass spec analyses suggested that HSV-2 glycoproteins B, C, and D (gB-2, gC-2, and gD-2) were antigens of the HSV-2 0ΔNLS vaccine (Fig. 5). Therefore, two-color Western blot analysis was applied to determine if antibodies from HSV-2 0ΔNLS-immunized mice bound the same HSV-2 proteins as IgG antibodies from rabbits immunized with gB-2, gC-2, or gD-2. IgG antibodies in rabbit gB-2 antiserum bound proteins in HSV-2 virions to 4.6-fold higher levels than total HSV-2-infected cell proteins (red bands in Fig. 6A). However, the IgG binding patterns of mouse 0ΔNLS antiserum revealed only weak overlap with the proteins bound by rabbit anti-gB-2 antiserum (Fig. 6A, merged signals in right blot). Hence, gB-2-specific antibodies were not clearly identifiable in this 0ΔNLS-immunized mouse by two-color Western blot. Likewise, the sera of n = 4 other HSV-2 0ΔNLS-immunized mice was similarly analyzed in two-color Western blots, and in all cases mouse 0ΔNLS antiserum did not contain high levels of IgG antibody that bound the same proteins as rabbit gB-2-specific antibodies (not shown).

An equivalent two-color Western blot was performed to assess the co-localization of IgG antibodies from HSV-2 0ΔNLS-immunized mice versus IgG from rabbit gC-2 antiserum (Fig. 6B). Again, two-color Western blot analysis failed to reveal clear evidence that gC-2 was a dominant target of the antibody response of HSV-2 0ΔNLS-immunized mice (Fig. 6B). In contrast, IgG antibodies in rabbit gD-2 antiserum bound an ~55 kDa protein in HSV-2 virions to 3.2-fold higher levels than total HSV-2-infected cell proteins (red bands in Fig. 6C), and this pattern of rabbit anti-gD-2 reactivity clearly overlapped with a target of mouse IgG antibodies in 0ΔNLS antiserum (Fig. 6C). Hence, the serum of this HSV-2 0ΔNLS-immunized mouse appeared to contained a sizable population of IgG antibodies against gD-2. Likewise, the serum of n = 4 other HSV-2 0ΔNLS-immunized mice contained IgG antibodies that co-localized to varying extents with rabbit IgG in gD-2 antiserum (not shown).

Although gD-2 was the 9^th^ most dominant source of viral peptides in IP-mass spec analyses (Fig. 5), gD-2 was readily confirmed as a *bona fide* antibody-generating protein of HSV-2 0ΔNLS by two-color Western blot analysis (Fig. 6C). Although gB-2-peptides were detected 3 times more frequently than gD-2-peptides in IP-mass spec analyses (Fig. 5), two-color Western blot analysis suggested that gB-2-antibodies were far less prevalent in HSV-2 0ΔNLS antiserum (Fig. 6A vs 6C). These results brought into focus the acute need for confirmatory testing to differentiate (1) immunoprecipitated HSV-2 proteins that might be false positives versus (2) *bona fide* antibody-generating proteins of the live HSV-2 0ΔNLS vaccine.

### Confirmation Method 2: antigen-deleted HSV-2 deletion mutants

HSV-2 ΔgD was used to explore the potential of an antigen-deleted HSV-2 mutant virus to empirically verify the contribution of gD-specific antibodies to the total population of HSV-2-specific antibodies present in gD-2- or HSV-2 0ΔNLS-immunized mice. To this end, serum IgG from immunized mice was compared for its ability to bind proteins in cells inoculated with ***i.*** wild-type HSV-1, ***ii.*** HSV-1 ΔgD virus, ***iii.*** wild-type HSV-2, or ***iv.*** HSV-2 ΔgD virus.

IgG in mouse gD-2 antiserum reacted exclusively with an ~55 kDa protein expressed in wild-type HSV-1 or HSV-2-infected cells, but which was absent in cells inoculated with HSV-1 or HSV-2 ΔgD viruses (band 8 in Fig. 7A). Recombinant gD-1 subunit vaccines elicit an antibody response that extensively cross-reacts with HSV-2 [30, 41], and reciprocally antibodies raised against recombinant gD-2 extensively cross-reacted with HSV-1 gD (Fig. 7A). This is atypical of the serotype-specific antibody responses to wild-type HSV-1 vsersus HSV-2 [37, 38]. Hence, although IgG in mouse 0ΔNLS antiserum bound at least 9 protein bands in HSV-2 infected cell, antibodies in HSV-2 0ΔNLS antiserum only weakly bound the homologous proteins expressed by HSV-1 (Fig. 7B). Likewise, IgG in mouse 0ΔNLS antiserum bound at least 6 protein species expressed by HSV-2 ΔgD virus albeit at lower levels (Fig. 7B).

Flow cytometry was used to compare the efficiency of IgG binding to fixed and permeabilized test cells that were ***i.*** uninfected (UI) or were inoculated with ***ii.*** HSV-2 ΔgD or ***iii.*** wild-type HSV-2 MS. A mixture of these cell populations was combined with 1:6,000 dilutions of mouse serum. UI cells, HSV-2 ΔgD^+^ cells, and HSV-2 MS^+^ cells were distinguished from one another by differential labeling with no, low, or high concentrations of the green fluorophore CFSE (Fig. 8A–D). Naïve mouse serum defined the background level of IgG antibody binding to test cells (mean fluorescent intensity [MFI] 3,500 to 5,700; Fig. 8A, 8E). IgG antibodies in gD-2-antisera exhibited a similarly low background binding to UI cells (MFI ~4,650), but bound HSV-2 MS^+^ cells to 15-fold higher levels (MFI ~68,200; Fig. 8B). IgG antibodies in gD-2-antisera failed to bind HSV-2 ΔgD^+^ cells to levels above UI cells, hence formally demonstrating that all virus-specific antibodies in gD-2-antisera were specific for the deleted gD antigen (Fig. 8B, 8E). In contrast, IgG antibodies in the sera of HSV-2 0ΔNLS-immunized mice bound HSV-2 ΔgD^+^ cells and wild-type HSV-2 MS^+^ cells to high and nearly equivalent levels that were well above the background of UI cells; hence, the majority of virus-specific antibodies in 0ΔNLS-antisera appeared to be specific for HSV-2 proteins other than gD (Fig. 8C, 8E). Likewise, sera of MS+ACV-immunized mice exhibited a similar pattern of antibody reactivity against HSV-2 MS^+^ cells versus HSV-2 ΔgD^+^ cells (Fig. 8D, 8E). Hence, both Western blot and flow cytometry tests (Figs. 7, 8) formally demonstrated that the majority of virus-specific antibodies in HSV-2 0ΔNLS-immunized mice were directed against viral antigens other than gD-2.

### Confirmation Method 3: cell lines expressing epitope-tagged HSV-2 antigens

Several experiments suggested that recipients of the live HSV-2 0ΔNLS vaccine mounted a greater antibody response to HSV-2’s RR-1 protein relative to gD-2 (Figs. 1–5; Proteins 3 and 8 in Table 1). However, it was possible that the apparent “dominance” of RR-1 over gD-2 in such experiments simply reflected the abundance of RR-1 protein in HSV-2 infected cell extracts relative to gD-2. The following experiment was performed to address this caveat, and formally compare the abundance of gD-2-, ICP8-, RR-1-, and VP5-specific antibodies in HSV-2 0ΔNLS antiserum in an experiment where all test antigens were present in equivalent amounts.

To this end, Vero cell lines were constructed that stably expressed FLAG-tagged variants of HSV-2 gD, ICP8, RR-1, and VP5 proteins where the 8-amino-acid FLAG epitope (DYKDDDDK) was placed at the C-terminus of each recombinant protein. Mouse sera were tested against Western blots containing lysates harvested from UI Vero cells, HSV-2-infected Vero cells, or Vero cells expressing individual, FLAG-tagged HSV-2 proteins (Fig. 9).

Naïve serum did not exhibit specific reactivity against viral proteins (Fig. 9A, left panel). Re-probing the same blot with anti-FLAG antibody verified that ICP8-FLAG, RR-1-FLAG, and VP5-FLAG were present in equivalent amounts whereas twice as much gD-FLAG was, by design, present on the blot (Fig. 9A, right panel). In n = 3 naïve sera tested, antibodies against FLAG-tagged gD, ICP8, RR-1, or VP5 were consistently undetectable (Fig. 9B).

An equivalent approach was applied to the serum of gD-2-immunized mice (Fig. 9C). High levels of IgG bound HSV-2’s ~55 kDa gD-2 protein, as well as FLAG-tagged gD-2 (Fig. 9C, left panel). Blots were re-probed with anti-FLAG antibody to measure the relative amounts of FLAG-tagged gD, ICP8, RR-1, and VP5 targets on each blot (Fig. 9C, right panel). After normalizing for differences in each FLAG-tagged target, the serum of n = 3 gD-2-immunized mice had levels of antibodies against gD that were 240 ± 100-fold above background, whereas antibodies against ICP8, RR-1, or VP5 were consistently undetectable (Fig. 9D).

HSV-2 0ΔNLS antiserum contained IgG antibodies that reacted with numerous viral proteins in HSV-2-infected cells whose MWs ranged from 35 to 160 kDa (Fig. 9E, left panel). This particular HSV-2 0ΔNLS antiserum contained antibodies that reacted with FLAG-tagged variants of gD, ICP8, RR-1, and VP5 (Fig. 9E, left panel). Western blots incubated with a total of n = 6 0ΔNLS antiserum samples indicated that the relative levels of serum antibody to these four HSV-2 antigens were heterogeneous (S4 Fig.). For example, in HSV-2 0ΔNLS-immunized mice 2, 4, and 6, ICP8-specific antibodies were more abundant than gD-specific antibodies, whereas in mice 1, 3, and 5, the reciprocal was true (S4 Fig.). In contrast, when normalized to account for the 2-fold excess of gD-FLAG on each blot, RR-1-specific antibodies were more abundant than gD-specific antibodies in 5 of 6 0ΔNLS-immunized mice (S4 Fig.). While VP5-specific antibodies were detected in 5 of 6 0ΔNLS-immunized mice, the level of VP5-specific antibody that bound VP5-FLAG was consistently near the lower limit of detection (S4 Fig.).

All 0ΔNLS antiserum-stained Western blots were re-probed with anti-FLAG antibody to quantify the amount of FLAG-tagged gD, ICP8, RR-1, and VP5 targets on each blot (Fig. 9E, right panel). After normalizing for differences in target abundance, n = 6 HSV-2 0ΔNLS antiserum samples contained RR-1-specific antibodies at levels that were 225 ± 50-fold above the background, and were an average ~5-fold more abundant than gD-specific antibodies (Fig. 9F). These same n = 6 HSV-2 0ΔNLS antiserum samples contained statistically indistinguishable levels of gD- and ICP8-specific antibodies, which were 44 ± 15- and 28 ± 18-fold, respectively, above background (Fig. 9F). In contrast, VP5-specific antibodies in 0ΔNLS antiserum were only detected at levels that were 3 ± 1-fold above background (Fig. 9F). Collectively, these results verified that gD, ICP8, RR-1, and VP5 are all *bona fide* antibody-generating proteins of the live HSV-2 0ΔNLS vaccine, and suggested that among these four HSV-2 proteins, RR-1 is typically the most dominant antigen of the HSV-2 0ΔNLS vaccine.

## Discussion

### Perspective

Over 14,000 individuals have been enrolled in U.S. clinical trials of glycoprotein subunit vaccines [6, 7, 8, 9, 10, 11], whereas n = 0 individuals have participated in U.S. clinical trials of a live, replication-competent HSV-2 vaccine [23]. Given that glycoprotein subunit vaccines have failed to prevent or reduce the symptoms of HSV-2 genital herpes in trials dating back to the late-1980s [6, 7, 8, 9, 10, 11], perhaps it is time to consider the logical alternative of a live HSV-2 vaccine. Although subunit vaccines are incredibly safe, they are based on <2% of HSV-2’s proteome. When >98% of HSV-2’s proteome is excluded from a vaccine, this raises the possibility that such vaccines may not engage the full repertoire of virus-specific B- and T-cells available to combat HSV-2 infection. The live HSV-2 0ΔNLS vaccine elicits up to 100-fold greater protective immunity against HSV-2 relative to a gD-2 subunit vaccine [16, 17], and this difference may, in part, be attributable to the fact that HSV-2 0ΔNLS expresses up to 99% of HSV-2’s proteome [23]. The results of the current study are consistent with this hypothesis, as recipients of the live HSV-2 0ΔNLS vaccine elicit an IgG antibody response against 9 to 19 different HSV-2 proteins.

### A live HSV-2 vaccine elicits an antibody response against capsid proteins, glycoproteins, infected cell proteins, and tegument proteins

HSV-2’s glycoproteins have been the focus of vaccine development efforts for 30 years [11, 41, 42, 43]. We initially expected to confirm that gD-2 and a combination of other glycoproteins were the dominant antigens of the live HSV-2 0ΔNLS vaccine. The data did not support this interpretation. Rather, Western Blot analyses indicated that many of the antibodies elicited by the HSV-2 0ΔNLS vaccine bound to viral proteins that were >110 kDa in size (Fig. 1, 2), and which were more abundant in virus-infected cells than virions (Fig. 4); most HSV-2 glycoproteins are <90 kDa in size and are enriched in virions. Initially, we were concerned these results were peculiar to the mouse model, but Western blot tests using ***i.*** serum from guinea pigs immunized with HSV-2 0ΔNLS or ***ii.*** human carriers of wild-type HSV-2 yielded similar results (data not shown).

IP-mass spec analysis of a HSV-2 0ΔNLS antiserum sample (i.e., 0ΔNLS-6 in S4 Fig.) suggested that HSV-2 infected cell proteins (e.g., RR-1, ICP8), capsid proteins (VP5 and VP22), tegument proteins (VP16 and VP1–2), and virion glycoproteins (gD-2) are potential antibody-generating components of the live HSV-2 0ΔNLS vaccine. Tests with individual FLAG-tagged HSV-2 antigens suggested that ICP8 and gD-2 are equally important as antibody-generating proteins of the live HSV-2 0ΔNLS vaccine (Fig. 9). In contrast, Western blot tests with total HSV-2 proteins and FLAG-tagged VP5 suggest that when the VP5 protein is denatured, it is a relatively minor HSV-2 antigen (Fig. 9). Of course, this leaves unanswered the question of whether (1) HSV-2’s major capsid protein, VP5, is a dominant antigen when its native conformation is maintained, or (2) VP5 is over-represented in IP-mass spec experiments (Fig. 5) for a variety of possible reasons. Finally, the HSV-2 RR-1 protein appears to elicit 4- to 5-fold higher levels of antibodies in 0ΔNLS antiserum relative to gD-2 and ICP8 (Fig. 9), and appears to be among the most dominant antibody-generating proteins of the HSV-2 0ΔNLS vaccine (Figs. 1–5). The status of other putative antigens of the HSV-2 0ΔNLS vaccine, as determined by IP-mass spec (Fig. 5), remains an open and unaddressed question that will require further study to resolve. Collectively, the results of this study raise important questions about how the majority of IgG antibodies, which do not appear to be directed against HSV-2 glycoproteins, contribute to 0ΔNLS vaccine-induced protection against HSV-2 genital herpes.

### RR-1 and ICP8: dominant HSV-2 antigens in several biological contexts

RR-1 and ICP8 emerged from these studies as two unexpectedly dominant antigens of the live HSV-2 0ΔNLS vaccine. Other lines of investigation suggest that HSV-2’s RR-1 and ICP8 proteins may be significant B- and T-cell immunogens. Investigators at the University of Washington have studied the CD4^+^ and CD8^+^ T-cell responses to HSV-2 in infected persons, and have concluded that RR-1 (UL39) and gB-2 (UL27) are dominant CD8^+^ T-cell immunogens [44]. Likewise RR-1 (UL39) and gD-2 are dominant CD4^+^ T-cell immunogens in symptomatic carriers of HSV-2 [45]. Intriguingly, asymptomatic carriers of HSV-2 develop CD4^+^ T-cell responses to a narrow range of HSV-2 proteins, yet RR-1 remains the most frequently targeted HSV-2 protein in such individuals [45]. ICP8 (UL29) is also a target of the CD4^+^ and CD8^+^ T-cell response to HSV-2, but is less dominant than RR-1 [44, 45].

In the 1970s and 1980s, investigators noted an ~160 kDa viral antigen dubbed “AG-4” that was precipitated by the serum of >90% of persons infected with HSV-2 [39, 46]. Individuals with asymptomatic HSV-2 infections also possessed high levels of IgG antibody against AG-4 [47, 48]. Further studies revealed that AG-4 consisted of a complex of HSV-2 RR-1 (UL39), RR-2 (UL40), and ICP8 (UL29) [34, 40]. Investigation of the AG-4 antigen halted in the mid-1980s, and thus the potential of RR-1- and ICP8-specific antibodies to contribute to protective immunity against HSV-2 was not explored.

### Can RR-1 and ICP8 antibodies contribute to protective immunity against HSV-2?

Antibodies against HSV-2 glycoproteins are thought to be critical to protective immunity. In large part, this belief is based on an assumption that antibody-mediated neutralization of virions is the central mechanism by which antibodies contribute to antiviral immunity [27, 29, 49, 50]. While virion neutralization may be important, complement fixation and antibody-dependent cellular cytotoxicity (ADCC) may also be relevant in explaining how antibodies restrict HSV-2 spread *in vivo*. HSV-1 and HSV-2 encode a combination of immune-evasion receptors, gE-gI and gC, which respectively antagonize the pro-inflammatory effects of ***i.*** antigen-bound IgG [51] and the ***ii.*** central activator of the complement cascade, C3b [52]. The presence of such virus-encoded countermeasures suggests that antibodies and complement may be relevant to the immune response that restricts the spread of HSV-2 *in vivo*.

It is often presumed that antibodies directed against extracellular HSV-2 antigens, such as cell-surface glycoproteins, uniquely contribute to protective immunity [27, 29]. However, the distinction between *extracellular* versus *intracellular* antigens may be largely academic *in vivo*. HSV-2 is a cytopathic virus, which kills cultured cells within 12 hours and produces cytopathic effects *in vivo*. Once HSV-2-infected cells die *in vivo*, cell debris containing high levels of formerly intracellular antigens, such as RR-1 or ICP8, may efficiently be bound by antibodies. Hence, antibodies against RR-1 or ICP8 may activate the classical complement cascade, produce anaphylatoxins that promote local inflammation, and recruit NK-cells and T-cells to sites of complement fixation, which may lie directly adjacent to active sites of HSV-2 replication *in vivo*. Moreover, the RR-1 protein has been reported to possess a transmembrane segment and localize to the surface of HSV-2-infected cells [53]. Hence, it is possible that RR-1-specific antibodies contribute to complement fixation by binding the membranes of live, HSV-2 infected cells.

Protective non-neutralizing antibodies contribute to functional protection against several viruses including yellow fever virus [54], influenza virus [55], Ebola virus [56], West Nile Virus [57], vesicular stomatitis virus [58], vaccinia virus [59, 60], and HIV [61]. In contrast, the effector mechanisms that explain how antibodies contribute to host immunity to HSV-2 have not been systematically investigated. Therefore, further investigation will be required before we may distinguish whether RR-1- and ICP8-specific antibodies represent irrelevant decoy antibodies or non-neutralizing effectors that contribute to protective immunity against HSV-2.

### Caveats and limitations of the current study

Western blot analysis likely underestimates the number of antigens in the live HSV-2 0ΔNLS vaccine, as this method cannot detect antibodies directed against conformational epitopes of HSV-2 proteins (Figs. 1, 4). In contrast, IP-mass spec analysis may overestimate the number of antigens in the HSV-2 0ΔNLS vaccine, as some HSV-2 proteins pulled down by 0ΔNLS antiserum may not be *bona fide* targets of IgG antibodies, but rather may co-IP as part of larger protein complexes that are only incompletely denatured by IP buffer. Finally, a weak signal in a Western blot or IP reaction may simply reflect a lack of target protein in the sample rather than a lack of antibody. Most of the analyses performed in the current study are limited by this final caveat with the exception of the data presented in Fig. 9, where a monoclonal α-FLAG antibody was used to empirically verify that ample amounts of FLAG-tagged gD, ICP8, RR-1, and VP5 targets were available on Western blots.

Aside from these formal caveats, our experience with these methods suggests two common-sense considerations that constrain what may and may not be concluded from these analyses. ***First***, there is no one “best” HSV-2 antibody-evaluation approach that provides all the answers regarding the immunodominance heirarchy of antibody-generating proteins of a whole HSV-2 vaccine. Because each method has limitations, it is only through conducting a series of independent tests with 0ΔNLS antiserum that it became clear which viral proteins were dominant targets of the antibody response to HSV-2 0ΔNLS. In the current study, gD, ICP8, and RR-1 were three such antibody-generating proteins of HSV-2 0ΔNLS. Both Western blot and IP-mass spec analysis identified VP1–2 (Band 1 in Table 1) and VP5 as antigens of the HSV-2 0ΔNLS vaccine, but their quantitative significance remains unclear. ***Second***, it is unrealistic to suggest that a “representative serum antibody response” against a complex viral vaccine, like HSV-2 0ΔNLS, is a realistic outcome. Rather, individuals in a population of HSV-2 0ΔNLS vaccine recipients may mount an antibody response against 5 to 25 HSV-2 proteins, and the precise hierarchy of antigens targeted will naturally vary between vaccine recipients. Therefore, perhaps the largest caveat of this study is that the data obtained in a few mice provide only a very limited basis to appreciate how the complex antibody response to a live-attenuated HSV-2 vaccine would actually vary across a large population of human recipients.

Past studies of the antibody responses to the HSV-2 dl-529 or HSV-2 cJ2-D2 viral vaccines tested the *pooled sera* of vaccine recipients against Western blots of HSV-2 proteins [20, 24]. The experimental step of pooling serum negates all possibility of evaluating the individual-to-individual variance of the antibody response to a whole HSV-2 vaccine This is not just a theoretical concern; numerous studies demonstrate that the T-cell response to wild-type HSV-2 is heterogeneous [44, 45, 62]. Thus, an important metric of “immunodominance” of any HSV-2 protein is the frequency with which exposed individuals mount a T-cell response to a specific HSV-2 protein. Likewise, evaluation of the immunodominance of HSV-2 proteins as antibody-generators in the context of whole HSV-2 vaccines should consider the frequency of vaccine recipients whose antibody response targets a specific HSV-2 protein. By this metric, RR-1 appears to be among the most dominant antibody-generating proteins of the live HSV-2 0ΔNLS vaccine (S4 Fig.).

### Conclusion

Many synthetic HSV-2 vaccines that contain <2% of HSV-2’s proteome have been proposed and/or advanced to clinical trials over the past 25 years [6, 7, 8, 9, 10, 11, 26, 63, 64, 65, 66]. Relative to the best studied of these approaches, gD-2 based vaccines, the live HSV-2 0ΔNLS vaccine offers: ***1.*** ~100-fold greater antigenic breadth [23]; ***2.*** an ~40-fold greater pan-HSV-2 IgG antibody response [16]; ***3.*** ~100-fold greater protection against HSV-2 shedding after vaginal challenge [16, 17]; and ***4.*** the potential to elicit an adaptive immune response against >10 HSV-2 proteins (Figs. 1, 3, 5). Questions remain about the safety of a live HSV-2 *ICP0* ^-^ mutant vaccine (that is attenuated in SCID hosts [14, 15]). However, we note that 20 million people continue to be newly infected with wild-type HSV-2 each year we lack a vaccine. In light of the relative risks of these two options, perhaps it is time to explore the potential of a live-attenuated HSV-2 vaccine to stop the spread of genital herpes in the human population.

## Materials and Methods

### Ethics Statement

Mice were handled in accordance with the National Institutes of Health Guide for the Care and Use of Laboratory Animals. This study was approved by the Southern Illinois University School of Medicine Laboratory Animal Care and Use Committee, and was performed as described under approved protocol 205–08–019.

### Cells, viruses, antibodies, and drug treatments

Vero cells (American Type Culture Collection, Manassas, VA), glycoprotein D (gD)-complementing VD60 cells, and V15-D1 cells were maintained in Dulbecco’s Modified Eagle’s Medium (DMEM) supplemented with 5% fetal bovine serum (FBS), 100 U/ml penicillin G, and 100 mg/ml streptomycin hereafter referred to as complete DMEM. VD60 cells were kindly provided by David Johnson (Oregon Health & Science University, Portland, OR) [67]. V15-D1 cells were kindly provided by Patricia Spear (Northwestern University School of Medicine) [68]. ICP0-complementing L7 cells were kindly provided by Neal Deluca (Univ. of Pittsburgh [69]).

Vero cell lines that stably express FLAG-tagged variants of HSV-2’s glycoprotein D, ICP8, RR-1 and VP5 will be the topic of a forthcoming manuscript (unpublished data of Andrew Wilber and William Halford), and we defer complete characterization of these cell lines until this study. However, we briefly describe these cells, which were used in the final experiment of this study. A novel gene-delivery system was created based on a *Sleeping Beauty* transposable element that bears HSV-1’s bidirectional ICP0-L/ST promoter. On the right side of the promoter, which normally drives L/ST transcription [70], was placed a genetic selection marker that encodes a truncated nerve growth factor receptor (tNGFR) [71]. On the left side of the ICP0 promoter, which normally drives ICP0 mRNA transcription, was placed the coding sequences for gD-FLAG, ICP8-FLAG, RR-1-FLAG, or VP5-FLAG. Finally, the ICP0 promoter construct was modified to contain (1) two Tet-operators immediately downstream of the TATA box where ICP0 mRNA transcription initiates to make mRNA synthesis TetR-repressible [72] and (2) two Sleeping Beauty direct repeats were placed at the left and right ends of the construct [73]. The presence of these direct repeats allowed the Sleeping Beauty transposase to efficiently transfer (transpose) the ICP0 promoter-gene expression cassette into the chromosomes of transfected cells, such that 1–3% of transiently transfected cells became stable cell lines within the first 48 hours after transfection [74]. Parent Vero cells were co-transfected with three plasmids: (1) a Sleeping Beauty transposable gene expression cassette that co-expresses a selectable tNGFR marker and individual FLAG-tagged HSV-2 protein; (2) a Sleeping Beauty transposable CAGS promoter driving expression of the Tet Repressor and a puromycin selection marker from an IRES-based bicistronic transcript; and (3) a plasmid that transiently expressed the Sleeping Beauty transposase. Following transient transfection, puromycin selection for stably transfected cells was initiated at 48 hours post-transfection and was maintained for 3 weeks at which time fluorescence-activated cell sorting (FACS) was used to isolate a >99% pure population of tNGFR^+^ cells, which were cultured in the continued presence of puromycin.

Wild-type HSV-1 KOS and wild-type HSV-2 MS (American Type Culture Collection) were propagated and titered in Vero cells cultured with complete DMEM. The HSV-2 *gD* ^-^ mutant virus, HSV-2 ΔgD-BAC, was kindly provided by Falko Schmeisser (Food and Drug Administration; Bethesda, MD) [75], and was propagated and titered in (gD)-complementing VD60 cells. The HSV-1 *gD* ^-^ mutant virus, KOS-gD6, was kindly provided by Patricia Spear (Northwestern University School of Medicine), and was propagated and titered in (gD)-complementing V15-D1 cells [68]. The HSV-2 *ICP0* ^-^ mutant viruses, HSV-2 0ΔNLS and HSV-2 0ΔRING, have been previously described [15], and were propagated on L7 cells.

Rabbit polyclonal antibodies against HSV-2 glycoproteins B, C, and D (R68; R64; and R8, respectively) were kindly provided by Gary Cohen and Roselyn Eisenburg (University of Pennsylvania). Rabbit polyclonal anti-GFP antibody was obtained from Clontech Laboratories Inc. (Mountain View, CA). Mouse monoclonal anti-FLAG M2 antibody was obtained from Sigma-Aldrich (St Louis, MO).

Cycloheximide-release experiments to evaluate the kinetics of HSV-2 antigen expression were performed, as follows. Monolayer cultures of Vero cells were treated with 200 µM cycloheximide (Acros Organics, NJ) for 30 minutes prior to HSV-2 inoculation, and were inoculated with 5 pfu per cell of HSV-2 MS or HSV-2 0ΔRING, and were incubated in the presence of CHX to allow viral immediate-early mRNA to accumulate. After 10 hours, cycloheximide was removed, rinsed three times, and replaced with DMEM containing ***i.*** no drug, ***ii.*** 10 µg/ml actinomycin D (Amresco, Solon, OH), or ***iii.*** 300 µM acyclovir (Sigma-Aldrich Corp, St Louis, MO) and cultures were incubated an additional 7 hours prior to protein harvest.

### Western blot analysis

Protein lysates from uninfected Vero cells, HSV-infected Vero cells, or sucrose-purified HSV-2 virions were harvested using mammalian protein extraction reagent (Thermo Scientific, Rockford, IL) supplemented with 1M dithiotreitol and Halt protease inhibitor cocktail (Thermo Scientific). Similarly, protein lysates were harvested from Vero cells that individually expressed gD-FLAG, ICP8-FLAG, RR-1-FLAG, or VP5-FLAG at 24 hours post-induction of the ICP0 promoter. The ICP0 promoter was induced by (1) de-repression of the Tet Repressor within the ICP0 promoter with 3 μM doxycycline and (2) treatment with 30 pfu/cell of a VP16-expressing adenovirus vector [76]. After heat denaturation, 12 µg of uninfected total Vero cell protein, total HSV-infected Vero cell protein, PageRuler Plus MW markers (Thermo Scientific), and/or 2.5 µg sucrose-purified HSV-2 virions were resolved in an 8% denaturing polyacrylamide gel, and were transferred to nitrocellulose membranes. Protein blots were blocked in phosphate-buffered saline (PBS) containing 5% nonfat dry milk and were incubated overnight at 4°C in PBS + 0.1% Tween-20 (PBS-T) + 5% nonfat dry milk containing a 1:20,000 dilution of primary mouse serum and/or a 1:10,000 dilution of rabbit antisera against GFP, the FLAG epitope, or specific HSV-2 proteins. Following incubation with primary antibodies, membranes were washed four times with PBS-T and were incubated with secondary antibodies diluted 1:20,000 in PBS-T + 5% nonfat dry milk. The secondary antibodies used were goat anti-mouse IgG and/or goat anti-rabbit IgG conjugated to the infrared fluorescent dyes IRDye 680LT and IRDye 800CW, respectively (LI-COR Bioscience, Lincoln, NE). Protein blots were washed three times in PBS-T, rinsed in PBS to remove residual Tween-20, and were scanned for two-color fluorescence using the Odyssey Infrared imaging system (LI-COR Bioscience). Data were analyzed using Odyssey application software version 3.0.16 (LI-COR Bioscience).

### Densitometric analysis of Western blots

Densitometric analysis of IgG antibody binding was performed using the “Lane Profile” and “Report” features of Odyssey Application Software version 3.0.16 (LI-COR Bioscience). The “Report” feature was used to quantify the intensity of antibody banding within specific regions (rectangles) relative to identically-sized, background control regions.

The “Line Profile” feature was used to generate x-, y- data sets that were exported to Microsoft Excel, and which were used to generate the parallel tracks of IgG antibody binding to HSV-2 proteins in Western blots incubated with the sera of multiple animals per immunization group (Fig 2A, 2B). Each x-, y-datum pair in a data set corresponded to the antibody binding intensity (y) at a specific distance (x) from the origin of protein migration. Protein MW markers provided a basis to normalize x-axis distances across blots such that the x-axes of multiple blots could be equivalently scaled. The equation p = *a* ● *e* ^*k* ● *x*^ was used to describe p, predicted log (protein size), as a function of distance migrated, x, on the Western blot where ‘a’ and ‘k’ were constants that dictated the shape of the standard curve. The method of least squares and the “Solver” tool in Microsoft Excel was used to optimize the values of k and a, such that the difference between p, predicted log (protein size), and known log (protein size) of MW markers was minimized between 35 and 130 kDa. Regression analysis indicated that the resulting equations accurately described protein sizes between 35 and 130 kDa (r^2^ > 0.995).

At MWs above 150 kDa, a simple y = mx + b equation was used to crudely approximate the MW of proteins based on the observed difference in the distance migrated between the 130 and 250 kDa protein markers. Therefore, the reliability of size estimates of proteins above 150 kDa is limited. The data presented in Fig. 2 reports the intensity of IgG antibody binding as a function of the predicted size of HSV-2 proteins, as calculated by the above methods.

### Purification of HSV-2 virions

Monolayer cultures of Vero cells were established in complete DMEM in 100-mm dishes and were inoculated with 0.005 pfu per cell of HSV-2 MS, and dishes were frozen at -80ºC when cytopathic effect was complete at 72 hours p.i. Fifteen 100-mm dishes of cells were thawed, cell debris was removed by centrifugation at 2000xg for 5 minutes, and ~90 ml clarified supernatant (15 ml per tube) was loaded onto six discontinous sucrose gradients of 15% sucrose, 35% sucrose, and 60% sucrose. Gradients were centrifuged in a Beckman SW27 rotor at 25,000 rpm for 3 hours, and ten 1-ml fractions were collected from the interface between the 35% and 60% sucrose layers after piercing the bottom of each tube with a 23-g needle. Using a refractometer (Atago Ltd., Tokyo, Japan), the ~4 ml worth of fractions whose refractive indexes corresponded to the 35—60% sucrose interface were retained and pooled. Thus, the virion-rich interface from all six centrifuge tubes was collected, pooled, diluted 1:2 with PBS, and distributed onto two centrifuge tubes containing new discontinuous gradients of 15%, 35%, and 60% sucrose. The second pair of gradients was similarly centrifuged in a Beckman SW27 rotor, and the 35–60% sucrose interface was again collected as described above. The double-purified HSV-2 virions contained in these pooled fractions were diluted 1:2 in PBS, and were concentrated by ultracentrifugation in a Beckman SW50 rotor at 50,000 rpm for 30 minutes. The resulting pellets of purified HSV-2 virions were resuspended in mammalian protein extraction reagent (Thermo Scientific) supplemented with 1M dithiotreitol and Halt protease inhibitor cocktail (Thermo Scientific) and stored at -80ºC until used in Western blots.

### Measurement of IgG antibody-binding to HSV-2 MS^+^ cells versus HSV-2 ΔgD^+^ cells

Single-cell suspensions of uninfected or HSV-2 infected Vero cells were generated for antibody staining and flow cytometric analysis, as follows. Nine monolayer cultures of Vero cells were established in 100-mm dishes in complete DMEM, and six hours later were mock-inoculated or inoculated with 5 pfu per cell of HSV-2 MS or HSV-2 ΔgD (n = 3 dishes per group). At 12 hours p.i., culture medium was removed, cells were gently rinsed with PBS (such that they remained attached), and cells were dispersed into single cell suspensions using a P-1000 pipettor and 3 ml PBS + 5 mM EDTA per dish.

Carboxyfluorescein succinimidyl ester (CFSE) was used to differentially label ***i.*** uninfected (UI) cells; ***ii.*** cells inoculated with HSV-2 ΔgD virus; versus ***iii.*** cells inoculated with wild-type HSV-2 MS. Specifically, this was achieved by spiking the PBS + 5 mM EDTA solutions used to disperse UI cells, ΔgD^+^ cells, and MS^+^ cells with 0, 0.45, or 6.0 µM of CFSE, respectively. Following cell dispersal and CFSE labeling, cells were transferred to 50-ml conicals and 20 ml PBS + 3% fetal bovine serum (PBS-F) was added to halt the CFSE labeling reaction. Cells were centrifuged at 200xg for 5 minutes to pellet cells, rinsed with 20 ml PBS-F, and centrifuged again. After these rinses, each population of Vero cells was resuspended in 4 ml PBS followed by 8 ml of 37% formaldehyde to fix cells and fix protein-bound CFSE. After 20 minutes, cells were centrifuged and re-suspended in 12 ml 90% methanol to permeabilize cells. After 10 minutes, cell were centrifuged, re-suspended in PBS-F, and cell clumps were removed from fixed and permeabilized cells by sequentially passing each population through 40-µm nylon mesh (BD Biosciences, San Jose, CA) and a 25-g needle.

Fixed and permeabilized cells were counted on a hemacytometer, and a 3-population test sample was created by combining equivalent numbers of UI cells, HSV-2 ΔgD^+^ cells, and HSV-2 MS^+^ cells. Cells were centrifuged and re-suspended in PBS-F-Ig blocking solution (i.e. PBS-F supplemented with 20 µg/ml each donkey γ-globulin, goat γ-globulin, and human γ-globulin; Jackson Immunoresearch Laboratories, Inc., West Grove, PA). Aliquots of this 3-population test sample (400 µl containing 750,000 cells) were distributed into 1.7-ml microfuge tubes, and 2 µl of 1:30-diluted mouse serum was added to achieve a final dilution of 1:6,000.

IgG antibody was allowed 2 hours to bind HSV-2-infected cells while tubes turned on a LabQuake rotisserie rotator (Barnstead International, Dubuque, IA). Mouse serum was removed rinsed from cells by two sequential 1.25-ml washes with PBS-F using a swinging bucket rotor to pellet cells following each rinse. IgG antibody-binding to cells was detected by adding a 1:1,000-dilution of the secondary antibody, allophycocyanin (APC)-conjugated goat anti-mouse IgG Fc-fragment (Jackson Immunoresearch Laboratories, Inc.). After a 1-hour incubation, excess secondary antibody was removed by three sequential washes with 1.25 ml of PBS-F.

Cells were resuspended in 0.2 ml PBS-F and analyzed by two-color flow cytometry in the FL1 and FL4 channels of an Accuri C6 flow cytometer to detect CFSE and APC, respectively (Accuri Cytometers, Inc., Ann Arbor, MI). IgG levels binding to HSV-2 infected cells was calculated based on the difference in mean fluorescence intensity (MFI) of UI cells versus HSV-2^+^ cells (ΔMFI = MFI_HSV-2 −_MFI_UI_). Background fluorescence was defined as the average ΔMFI observed in 3-population test cells incubated with naïve mouse sera.

### Immunoprecipitation-mass spectrometry analysis

Two 100-mm dishes containing 10^7^ Vero cells were mock-inoculated and two dishes were inoculated with 5 pfu per cell of HSV-2 MS. At 12 hours p.i., Vero cells were washed with ice-cold phosphate-buffered saline and lysed in 0.5 ml of an IP buffer (50 mM Tris (pH 7.4), 150 mM sodium chloride, 2 mM EDTA, 1% NP40, and 1× Halt protease inhibitor cocktail) for 2 hours at 4°C on a rotisserie, cell debris was removed by centrifugation, and supernatants were pre-cleared by incubation with Protein A/G agarose beads (SantaCruz Biotechnology) for 30 minute at 4°C. In the first immunoprecipitation experiment (IP Exp 1), the pre-cleared supernatants were incubated with ***1.*** 10 µl normal mouse serum or ***2.*** 10 μl HSV-2 0ΔNLS antiserum. In the second immunoprecipitation experiment (IP Exp 2), the amount of mouse serum was reduced by 50% (5 μl serum per IP reaction) based on optimization experiments that revealed 1% serum yielded a better signal-to-noise ratio. In IP Exp 1 and 2, immune complexes were allowed 2 hours to form and were then incubated overnight with 30 μl of Protein A/G agarose beads (Santa Cruz Biotechonology) on a rotisserie rotator at 4ºC. Immunnocomplexes were washed four times with IP buffer, and were prepared for SDS-PAGE by boiling in 2× Laemmli loading buffer. Following electrophoresis on an 8% polyacrylamide gel, gels were stained with Coomassie Blue to visualize immunoprecipitated proteins.

In IP Exp 1, three bands of interest were excised and sent to the University of Arkansas Proteomics Core for MALDI-TOF mass spectrometry protein identification. In IP Exp 2, the entire lane of a polyacrylamide gel was excised from the gel, and sent to the University of Arkansas Proteomics Core where the lane was subdivided into 18 slices, which were each subjected to MALDI-TOF mass spectrometry protein identification. Mass spectrometry protein identification results were analyzed with Scaffold v4.0 (Proteome Software Inc, Portland, OR).

In the latter experiment, a total of 16,522 HSV-2 peptides were identified by mass spectrometry as being derived from 43 HSV-2 proteins. Eighty-nine percent of these peptides (14,729) were derived from 19 HSV-2 proteins that met four criteria that were applied to minimize false-positive results, and these were: (1) A HSV-2 protein was only considered a significant target of the IgG antibody in 0ΔNLS antiserum if it contributed >1% to the pool of peptides derived from “positive” HSV-2 proteins; hence, only HSV-2 proteins that yielded >147 peptides in immunoprecipitates were considered “positive;” (2) At least 30% of peptides must co-migrate in 3 consecutive gel slices at the correct MW of the identified viral protein (e.g., S3 Fig.); (3) At least 25% of the total HSV-2 protein must be represented in the identified peptides; and (4) at least 10 unique peptides must be detected per identified HSV-2 protein.

## Supporting Information

S1 Fig Western blot analysis to screen for candidate antibody-generating proteins of the live HSV-2 0ΔNLS vaccine: complete results. Western blots of (UI) uninfected Vero cells or cells inoculated with 2.5 pfu/cell of HSV-1 KOS or HSV-2 MS incubated with 1:20,000 dilutions of serum from **(A)** sera from n = 5 mock-immunized mice (naïve) or n = 5 mice per group immunized with **(B)** gD-2 + alum/MPL adjuvant, **(C)** the HSV-2 0ΔNLS (*ICP0* ^-^) mutant, or **(D)** an acyclovir-restrained HSV-2 MS infection (MS+ACV). Red diamonds (1–9) denote the positions of HSV-2 proteins most commonly targeted by mouse IgG antibodies.
(TIF)

S2 Fig Western blot analysis of purified HSV-2 virions segregates candidate HSV-2 0ΔNLS antigens into infected cell proteins versus virion proteins: complete results. Western blots of (UI) uninfected Vero cells, total HSV-2-infected cell proteins (MOI = 2.5), or sucrose-gradient-purified HSV-2 virions were incubated with 1:20,000 dilutions of sera from **(A)** n = 5 mock-immunized mice (naïve) or n = 5 mice per group immunized with **(B)** gD-2 + alum/MPL adjuvant, **(C)** HSV-2 0ΔNLS, or **(D)** an acyclovir-restrained HSV-2 MS infection (MS+ACV). Red diamonds (1–9) denote the positions of viral proteins in total HSV-2-infected cell samples commonly targeted by mouse IgG antibodies.
(TIF)

S3 Fig IP-mass spec experiment 2: slice-by-slice results for dominant antigens. The entire lane of a gel was analyzed by MALDI-TOF mass spectrometry after being cut into 18 equivalent sized slices (denoted by boxes 1–18). As shown in the graph, the number of peptide hits corresponding to VP1–2 peaked in the 3rd gel slice, which corresponded to a MW of ~200 to 250 kDa. In contrast, the peak of peptide hits corresponding to RR-1 and ICP8 peaked in the 6th gel slice, which corresponded to a MW of ~120 to 130 kDa. Importantly, 41 to 64% of the peptide hits against VP1–2, RR-1, and ICP8 were detected in three adjacent gel slices that corresponded to the expected MW of these proteins, which satisfied 1 of 4 criteria applied to minimize false-positives in this analysis.
(TIF)

S4 Fig Relative abundance of gD-, ICP8-, RR-1-, and VP5-specific antibodies in HSV-2 0ΔNLS antiserum: complete results. Western blots of (UI) uninfected Vero cells, cells inoculated with 2.5 pfu/cell of HSV-2 MS, or Vero cell lines that stably express the following, recombinant HSV-2 proteins: gD-FLAG, ICP8-FLAG, RR-1-FLAG, or VP5-FLAG incubated with 1:20,000 dilutions of serum from n = 6 mice immunized with the live HSV-2 0ΔNLS vaccine. Although not shown, all blots were rinsed and re-probed with mouse α-FLAG antibody to verify the relative amount of FLAG-tagged HSV-2 protein on each blot. These data form the basis for the quantitative results presented in Fig. 9F.
(TIF)
