# Supplementary figures and images for: Herpes Simplex Virus 2 (HSV-2) Infected Cell Proteins Are among the Most Dominant Antigens of a Live-Attenuated HSV-2 Vaccine
Source: PLoS One. 2015 Feb 6;10(2):e0116091. doi: 10.1371/journal.pone.0116091 (PMC4319894; doi:10.1371/journal.pone.0116091)

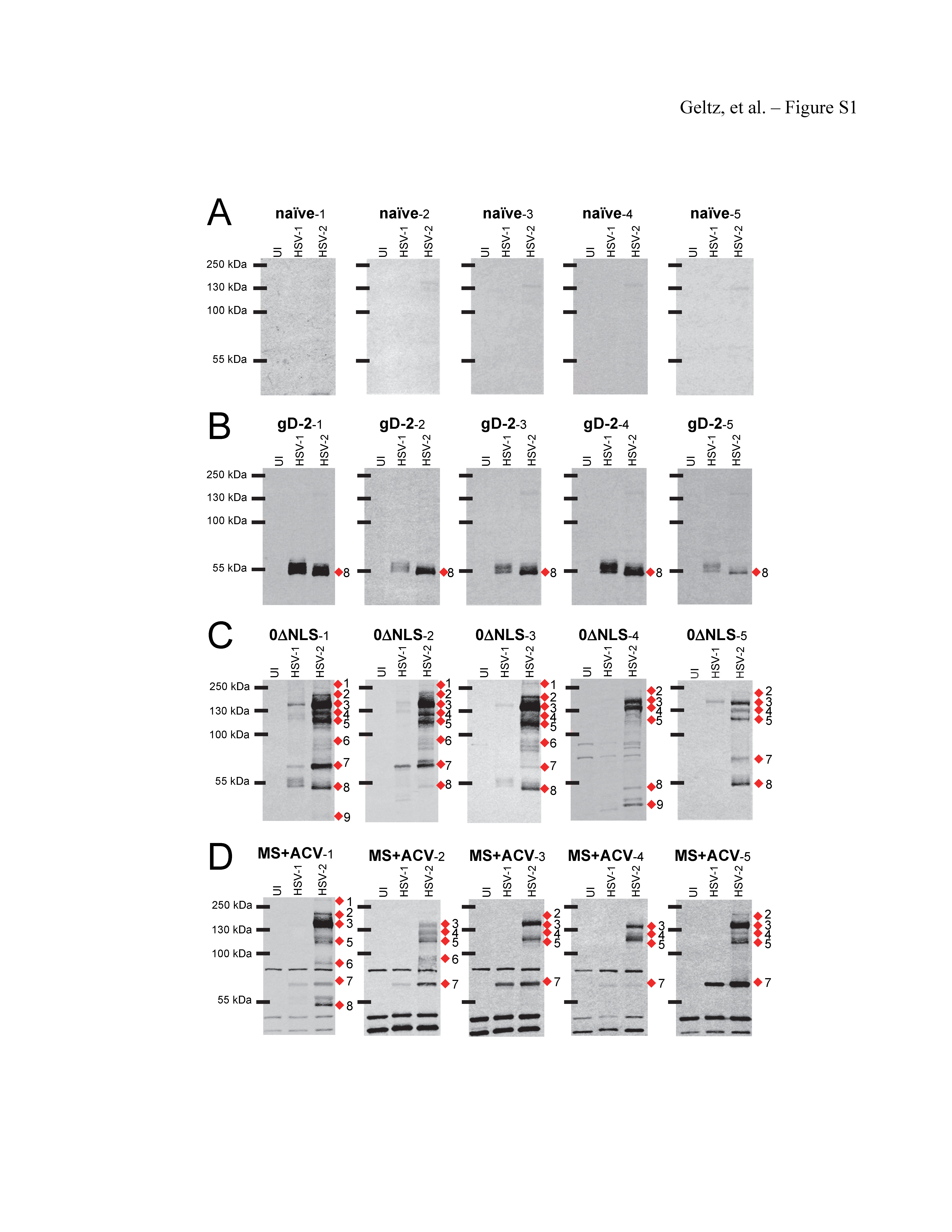

Supplement: S1 Fig — Western blots of (UI) uninfected Vero cells or cells inoculated with 2.5 pfu/cell of HSV-1 KOS or HSV-2 MS incubated with 1:20,000 dilutions of serum from (A) sera from n = 5 mock-immunized mice (naïve) or n = 5 mice per group immunized with (B) gD-2 + alum/MPL adjuvant, (C) the HSV-2 0ΔNLS (ICP0 -) mutant, or (D) an acyclovir-restrained HSV-2 MS infection (MS+ACV). Red diamonds (1–9) denote the positions of HSV-2 proteins most commonly targeted by mouse IgG antibodies. (TIF) [file pone.0116091.s001.tif]

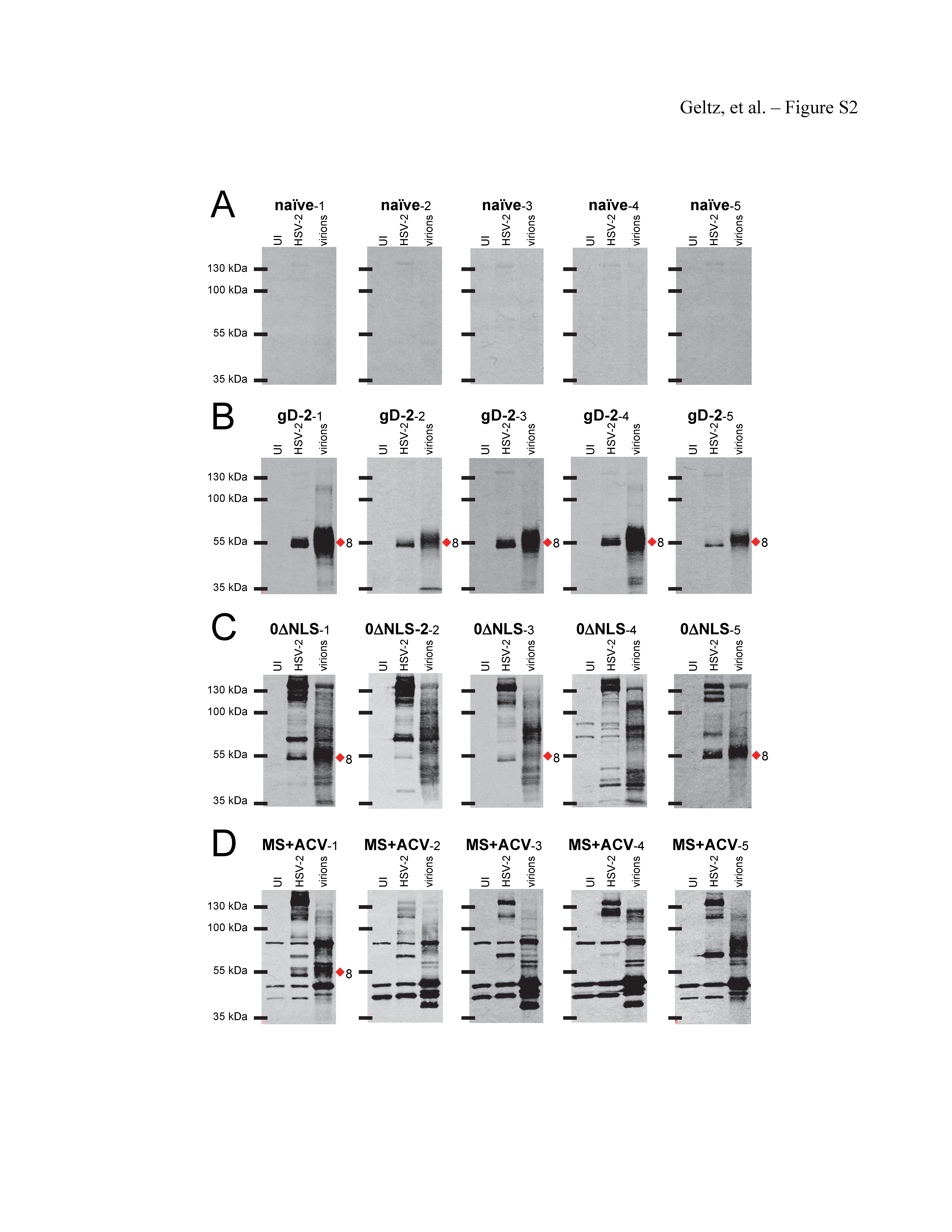

Supplement: S2 Fig — Western blots of (UI) uninfected Vero cells, total HSV-2-infected cell proteins (MOI = 2.5), or sucrose-gradient-purified HSV-2 virions were incubated with 1:20,000 dilutions of sera from (A) n = 5 mock-immunized mice (naïve) or n = 5 mice per group immunized with (B) gD-2 + alum/MPL adjuvant, (C) HSV-2 0ΔNLS, or (D) an acyclovir-restrained HSV-2 MS infection (MS+ACV). Red diamonds (1–9) denote the positions of viral proteins in total HSV-2-infected cell samples commonly targeted by mouse IgG antibodies. (TIF) [file pone.0116091.s002.tif]

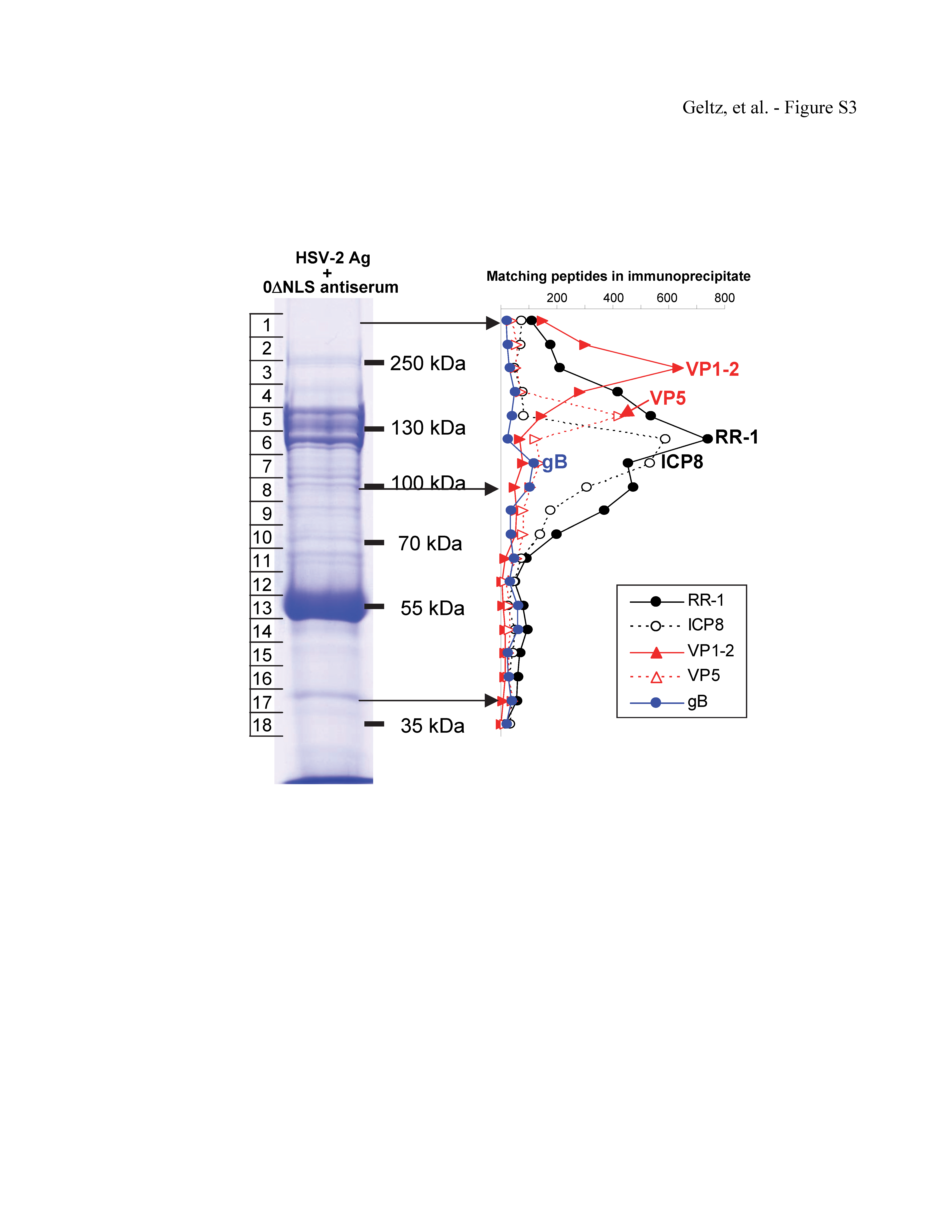

Supplement: S3 Fig — The entire lane of a gel was analyzed by MALDI-TOF mass spectrometry after being cut into 18 equivalent sized slices (denoted by boxes 1–18). As shown in the graph, the number of peptide hits corresponding to VP1–2 peaked in the 3rd gel slice, which corresponded to a MW of ~200 to 250 kDa. In contrast, the peak of peptide hits corresponding to RR-1 and ICP8 peaked in the 6th gel slice, which corresponded to a MW of ~120 to 130 kDa. Importantly, 41 to 64% of the peptide hits against VP1–2, RR-1, and ICP8 were detected in three adjacent gel slices that corresponded to the expected MW of these proteins, which satisfied 1 of 4 criteria applied to minimize false-positives in this analysis. (TIF) [file pone.0116091.s003.tif]

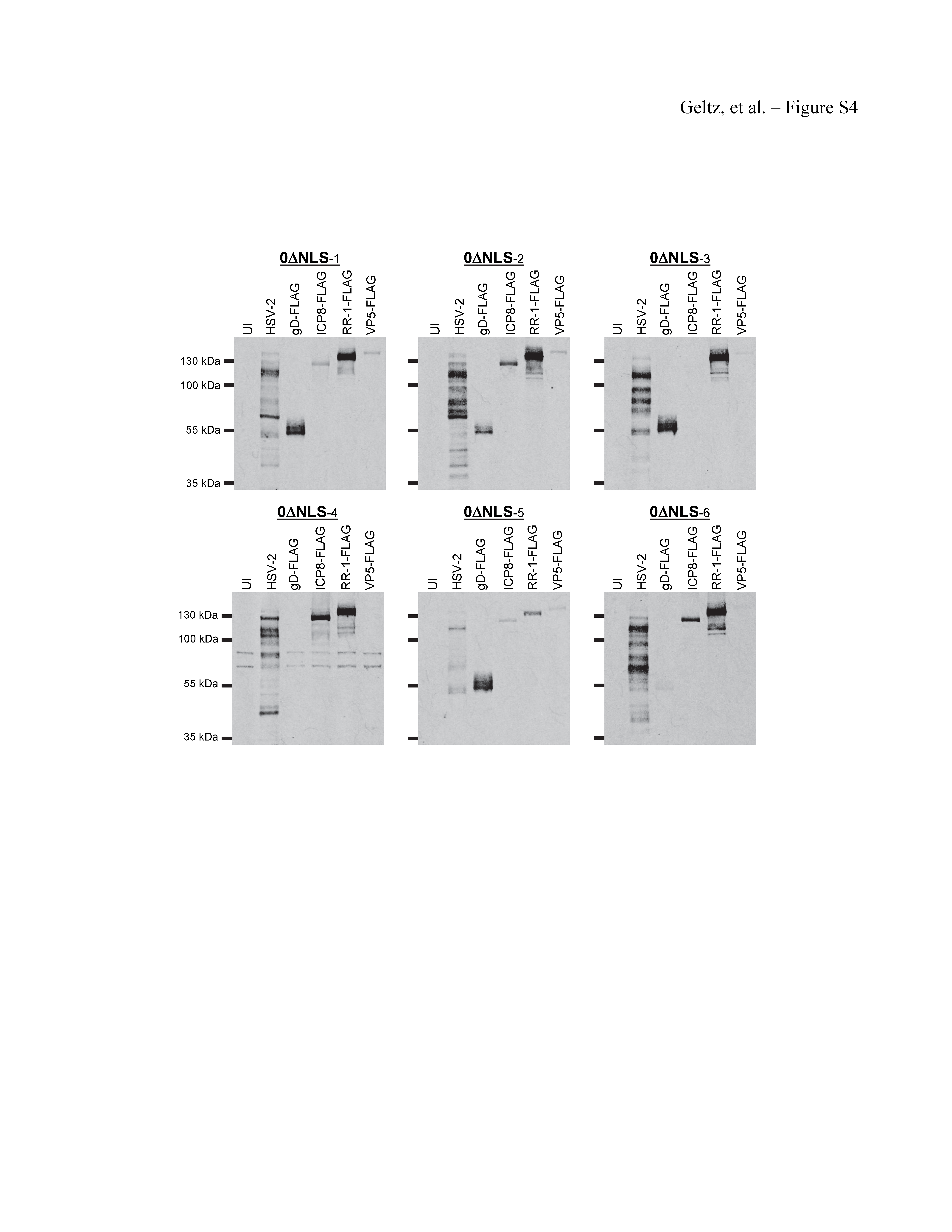

Supplement: S4 Fig — Western blots of (UI) uninfected Vero cells, cells inoculated with 2.5 pfu/cell of HSV-2 MS, or Vero cell lines that stably express the following, recombinant HSV-2 proteins: gD-FLAG, ICP8-FLAG, RR-1-FLAG, or VP5-FLAG incubated with 1:20,000 dilutions of serum from n = 6 mice immunized with the live HSV-2 0ΔNLS vaccine. Although not shown, all blots were rinsed and re-probed with mouse α-FLAG antibody to verify the relative amount of FLAG-tagged HSV-2 protein on each blot. These data form the basis for the quantitative results presented in Fig. 9F. (TIF) [file pone.0116091.s004.tif]
